# Fish Oil Supplementation Attenuates Offspring’s Neurodevelopmental Changes Induced by a Maternal High-Fat Diet in a Rat Model

**DOI:** 10.3390/nu17101741

**Published:** 2025-05-21

**Authors:** Yasna Muñoz, Heidy Kaune, Alexies Dagnino-Subiabre, Gonzalo Cruz, Jorge Toledo, Rodrigo Valenzuela, Renato Moraga, Luis Tabilo, Cristian Flores, Alfredo Muñoz, Nicolás Crisosto, Juan F. Montiel, Manuel Maliqueo

**Affiliations:** 1 Laboratory of Endocrinology and Metabolism, Department of Internal Medicine West Division, Faculty of Medicine, University of Chile, Santiago 8350499, Chile; yasnamunoz.nut@gmail.com (Y.M.); cristian.flores.ramirez@gmail.com (C.F.); nicolascrisostok@gmail.com (N.C.); 2 Laboratorio de Neurociencias Integradas, Centro de Investigación Biomédica, Facultad de Medicina, Universidad Diego Portales, Santiago 8370007, Chile; 3 Laboratorio de Reproducción, Centro de Investigación Biomédica, Facultad de Medicina, Universidad Diego Portales, Santiago 8370007, Chile; heidy.kaune@udp.cl; 4 Laboratory of Stress Neurobiology, Centro Interdisciplinario de Estudios en Salud, Faculty of Science, Universidad de Valparaíso, Valparaíso 2360102, Chile; alexies.dagnino@uv.cl (A.D.-S.); luis.antonio.tabilo@gmail.com (L.T.); alfredo.munoz@uv.cl (A.M.); 5 Laboratory of Reproductive and Metabolic Alterations, Center for Neurobiology and Integrative Physiopathology (CENFI), Institute of Physiology, Faculty of Sciences, Universidad de Valparaíso, Valparaíso 2360102, Chile; gonzalo.cruznec@gmail.com; 6 Advanced Scientific Equipment Network (REDECA), Faculty of Medicine, University of Chile, Santiago 8350499, Chile; jtoledo@uchile.cl (J.T.); renatomoraga@ug.uchile.cl (R.M.); 7 Department of Nutrition, Faculty of Medicine, University of Chile, Santiago 8350499, Chile; rvalenzuelab@uchile.cl; 8 Endocrinology Unit, Department of Medicine, Clínica Alemana de Santiago, Faculty of Medicine, Universidad del Desarrollo, Santiago 7650567, Chile

**Keywords:** brain cortex, development, neurological reflexes, high-fat diet, fish oil

## Abstract

**Background/Objectives:** A maternal high-fat diet (HFD) impairs brain structure in offspring. In turn, fish oil (FO) rich in *n*-3 polyunsaturated fatty acids (PUFAs) has neuroprotective effects. Therefore, we investigated whether maternal HFD exposure affected the neurological reflexes, neuron morphology, and *n*-3 PUFA levels in the cerebral cortex of the offspring and whether these effects were mitigated by maternal FO consumption. **Methods:** Female Sprague Dawley rats received a control diet (CD, 10% Kcal fat) or HFD (45% Kcal fat) five weeks before mating and throughout pregnancy and lactation. From mating, a subgroup of HFD was supplemented with 11.4% FO into the diet (HFD-FO). Neurological reflexes were evaluated from postnatal day (PND) 3 until PND20. Brains were removed at PND22 for neuron morphology analysis. Moreover, fatty acid composition and transcripts of genes encoding for factors associated with synapse transmission (SNAP-25), plasticity (BDNF), transport of DHA (MFSD2a), and inflammation (NF-κB and IL-1β) were quantified in prefrontal, motor, and auditory cortices. **Results:** FO diminished the effects of HFD on the number of thin and mushroom-shaped dendritic spines in the cerebral cortex in both sexes. It also reversed the HFD effects on the motor and auditory reflexes in female and male offspring, respectively. In males, FO up-regulated *Bdnf* transcript levels in the motor cortex compared with CD and HFD. In females, *n*-3 PUFAs were higher in HFD and HFD-FO than in CD in the auditory cortex. **Conclusions:** Our results highlight the protective role of maternal dietary *n*-3 PUFAs in counteracting the effects induced by HFD on the acquisition of neurological reflexes and neuronal morphology in the cerebral cortex of the offspring of both sexes.

## 1. Introduction

A maternal high-fat diet (HFD) and obesity have been associated with abnormal neurological development in the offspring [1,2]. In addition, animal studies have indicated that the effects of HFD are influenced by sex, with males being more affected than females [3]. In rats and mice, the consolidation of neurological reflexes during the first weeks of life represents a hallmark of neurodevelopment and reflects the maturation of brain structures [4,5]. It has been shown that maternal exposure to HFD has adverse effects on the brain volume and the processes of neurogenesis, synaptogenesis, migration, axonal myelination, and maturation of neuronal cells in the hippocampus, dentate gyrus, and amygdala in the offspring [1,6]. However, few studies have evaluated the effect of HFD on the cerebral cortex and the maturation of higher cortical networks, which are central to establishing normal neurodevelopment. The mechanisms behind these effects are not entirely known. Still, they are probably mediated by the alteration of the expression of the brain-derived neurotrophic factor (BDNF) [7], which regulates synaptic plasticity, neurogenesis, and neuronal survival, and by the expression of proteins involved in the synaptic transmission and long-term synaptic plasticity such as synaptosome-associated protein 25 (SNAP-25) [8].

It has been proposed that HFD and obesity alter the brain’s development through mechanisms that involve insulin resistance, systemic inflammation, and an imbalance in the diversity and community composition of the gut microbiome, namely, gut dysbiosis. Insulin resistance increases free fatty acids (FAs), saturated FAs, and *n*-6 PUFAs, promoting oxidative stress and neuroinflammation in different brain areas, leading to neurodevelopmental derangements [9,10]. These processes are activated by multiple signals that converge in the phosphorylation and nuclear translocation of nuclear factor kappa B (NF-κB), which induces the transcription of pro-inflammatory cytokines such as interleukin 1β (IL-1β) in neurons and microglia [11]. In turn, gut dysbiosis, induced by obesity and HFD, dysregulates a wide range of neuroactive substances, gut-derived hormones, and gut microbiota-derived metabolites that affect multiple homeostatic systems, including the gut–brain axis, which refers to the bidirectional communication between the gastrointestinal tract and the central nervous system [12].

The brain mainly comprises lipids, of which PUFAs from the *n*-3 and *n*-6 series play central roles in brain development due to their participation in brain neuronal membranes [13]. The *n*-6 PUFAs series can be enzymatically synthesized from its precursor, linoleic acid (C18:2), which is metabolized to arachidonic acid (C20:4, ARA). Nevertheless, the latter is mainly obtained from the diet and can be found abundantly in beef, lamb, and pork [14]. On the other hand, the *n*-3 PUFAs series are synthesized from α-linolenic acid (C18:3), producing eicosapentaenoic acid (C20:5, EPA) and subsequently docosahexaenoic acid (C22:6, DHA), which is the most abundant FA in the brain [15]. The primary dietary source of *n*-3 PUFAs is high-fat fish such as tuna, jack mackerel, and salmon [14], among others, and there are also available nutritional supplements that have EPA and DHA mainly based on FO and vegetable oils rich in ALA [15,16].

The *n*-3 PUFAs have anti-inflammatory effects and increase the neuronal membrane’s fluidity, optimizing the position and function of membrane proteins and modulating synaptic plasticity [2,17]. It has been described that the major brain uptake of *n*-3 PUFAs occurs in its esterified form, mainly lysophosphatidylcholine (LPC). Knockout mice for the major facilitator superfamily domain-containing protein 2 (Mfsd2a) exhibit a reduction in the total level of DHA in the brain, indicating that the uptake of DHA is mediated through this transporter in the brain [18]. Chronic treatment with FO and lard diets influences their mRNA and protein expression in young adult mice [19,20].

Maternal supplementation with *n*-3 PUFAs has shown protective effects against HFD-induced insulin resistance, mitigating oxidative stress and inflammation. Interestingly, *n*-3 PUFAs, especially DHA, participate in multiple neurodevelopmental events, including neuronal differentiation, synaptogenesis, and modulating neurotransmission through bioactive messengers [13,21,22,23]. Additionally, DHA is metabolized by 15-lipoxygenase to produce docosanoids such as resolvins and neuroprotectins that produce neuroprotection due to their anti-inflammatory and anti-apoptotic properties [24]. Moreover, *n*-3 PUFAs can modulate the abundance of beneficial gut bacteria, inhibit the production of proinflammatory mediators, and promote the production of anti-inflammatory mediators in the gut [25]. Even more, interventions based on FO have shown beneficial effects in the offspring, improving scores in sequential processing, simultaneous processing, nonverbal skills, and short-term memory [26,27,28,29,30,31].

Therefore, considering that the cerebral cortex has a critical role in the establishment of normal neurodevelopment, which can be affected by poor dietary quality, we aimed to evaluate in an experimental model in rats whether the supplementation with FO during pregnancy and lactation avoids the detrimental effects of HFD on the offspring’s neurological reflexes and neuronal morphology in the cerebral cortex.

## 2. Materials and Methods

**Animals:** Four-week-old female and male rats of the Sprague Dawley strain were obtained from the Center for Innovation in Biomedical Experimental Models of the Pontificia Universidad Católica de Chile. The rats were housed in the animal facility at the Laboratory of Stress Neurobiology, Universidad de Valparaíso, on a standard 12 h light and 12 h dark schedule (lights were turned on at 07:00 a.m.). During the whole experiment, food and water were available ad libitum. All animal experiments were conducted following the guidelines and approval (CBC14/2021) of the Bioethics Committee for Animal Research of the Faculty of Sciences of the Universidad de Valparaíso.

**Study design (Figure S1, supplementary):** From the sixth week, female rats were divided randomly into two experimental groups, which began to receive a control diet (CD, D12450H, Research Diet, New Brunswick, NJ, USA, 10% Kcal fat, *n* = 6) or HFD (D12451, Research Diet, New Brunswick, NJ, USA, 45% Kcal fat, *n* = 13) for five weeks (Table 1 shows the composition of the diets). Mating was performed during the night of proestrus in the fifth week. Pregnancy was determined by evaluating the presence of a vaginal plug after 8 h of contact with the male. The day following the presence of the vaginal plug was considered gestational day 0.5 (E0.5). After mating, the HFD group was divided into 2 groups; the first group was fed ad libitum with the same diet, the HFD group (*n* = 7), and the second group was fed ad libitum with HFD enriched with FO in 11.4% for 100 g of diet (HFD-FO, D21030416, Research Diet, New Brunswick, NJ, USA, 45% Kcal fat, *n* = 6). In the HFD-FO group, one dam died during gestation for a reason not related to the diet. The FA composition of FO is shown in Supplementary Table S1. Evaluation of neurodevelopmental reflexes was carried out from PND3 until PND18. Euthanasia was performed on PND22 through cardiac puncture under isoflurane anesthesia. Brains were weighed and stored in RNAlater^TM^ solution (stabilization and storage solution, Ambion, Inc., Austin, TX, USA) at −80 °C for gene expression and fatty acid analysis.

For morphological analysis, brains were kept in 4% PFA for 24 h, washed in 1× PBS, and stored in 1× PBS with 0.001% sodium azide at 4 °C.

The total numbers of brains per group were stored as follows: CD (16 male and 17 female brains in RNAlater^TM^ solution, 16 male and 16 female brains in PFA), HFD (17 male and 15 female brains in RNAlater^TM^ solution, 15 male and 17 female brains in PFA), and HFD-FO (13 male and 12 female brains in RNAlater^TM^ solution, 12 male and 13 female brains PFA).

**Evaluation of neurodevelopmental reflexes:** Neurodevelopmental reflexes, such as upper and lower grasp, turning, edge avoidance, gait, eye opening, hearing, posture, and correction, were measured simultaneously and under the same temperature, noise, and humidity conditions. From PND3 until PND20, all pups from each litter were assessed daily until reflex development was complete [4]. The analysis considered the progress in consolidating the reflexes mentioned above. The results are presented as percentages of the total litter on heat maps, where 100% corresponds to reflex gaining in all litter pups. In addition, the day on which the reflex should appear was plotted based on the article by Nguyen et al., except for the auditory reflex, which was assessed from PND10 onward but was plotted on the *Y*-axis at PND12 [4].

**Cerebral cortex sampling:** Brains from six males and six females at PND22 in CD, seven males and seven females in HFD, and five males and five females in HFD-FO were processed for morphological analysis. Brains were cut coronally to obtain a sampling of the brain’s anterior, medial, and posterior areas. Each rat used for this analysis belonged to a different litter. Serial coronal slices of the brain (each brain was cut by interleaving slices of 50 or 300 µm thickness) were made from anterior to posterior using a vibratome (VT1000, Leica, Deer Park, IL, USA).

Tissues stored at −80 °C were processed for FA and RNA analysis, generating five 2.5 mm thick slices for stereoscopic dissection of the prefrontal, motor, and auditory cortices for subsequent fatty acid analysis by gas chromatography and RNA by qPCR. In each slice, the prefrontal (bregma 2.16 to 1.86 mm), motor (bregma 1.20 to 0.9 mm), and auditory cortices (bregma −1.32 to −1.62 mm) were identified based on the references of the stereotaxic coordinates.

**Cell density analysis:** Anterior, medial, and posterior 50 µm brain sections were mounted on slides. They were left to dry in an oven at 37 °C for 24 h and then stained with Nissl for 10 min. Finally, they were dehydrated by an alcohol battery.

**Neuronal density analysis:** Brain sections of 50 µm from anterior, medial, and posterior areas were stained by free floating. Endogenous peroxidase was inactivated twice for 15 min at room temperature (RT) with 6% H_2_O_2_ in PBS. After three washes with PBS, the sections were immersed in blocking solution (Normal Horse Serum, 2.5%, VECTASTAIN Elite ABC, Vector Labs, Newark, CA, USA) for 1 h at RT and then incubated with NeuN polyclonal antibody (1:1000, PA5-78639, Thermo Fisher Scientific, Waltham, MA, USA) as the primary antibody for 2 h at RT. After incubation and subsequent washes with PBS, the brain sections were incubated for 1 h at RT with the secondary antibody (Horse anti-mouse/rabbit IgG, VECTASTAIN Elite ABC). After subsequent washes with PBS, sections were incubated with avidin-biotin-peroxidase (1:200, VECTASTAIN Elite ABC Reagent, R.T.U.) for 1 h at RT. After washing, staining was revealed with diaminobenzidine (1:1, VECTASTAIN Elite ABC). Finally, the sections were washed in distilled water, dehydrated, and mounted.

**Tissue Scanner:** The 50 μm thick brain slices were scanned using the VENTANA DP 200 slide scanner (Roche Diagnostics, Basel, Switzerland) through its software VENTANA DP 200 Scan Application (v1.1). Scans were performed with a 20× objective (NA 0.75, Nikon Plan Apo Lambda, Melville, NY, USA) and a 40× optical zoom. Eleven layers were scanned (1 μm spacing), and files were saved in TIF format.

**Image analysis:** Cell quantification was performed with QuPath (v0.4.0) using the Cell Detection tool. Annotations of 2000 × 400 μm were drawn, with the major axis encompassing all gray matter layers. Analysis was performed in the anterior, medial, and posterior areas of the brain. Annotations and detected cells were exported to Fiji (v2.90/1.53 t) and rotated with MorphoLibJ (v1.6.0), so that the depth of the cortex was oriented from left (0 μm) to right (2000 μm) [32]. Find Maxima from Fiji was used to transform the cells into 1 × 1 pixel objects, from which a tracing profile of a region of interest (ROI) of the image size was obtained. For the Neun antibody, the quantification was performed through image analysis using QuPath software (v0.4.0) [33]. Each brain region (anterior, middle, and posterior) was analyzed as a single image. Two 80,000 µm^2^ squares were drawn on the open image using the “Annotations” tool, on which the Cell Detection analysis was run.

**Morphological dendrites analyses:** Following the manufacturer’s protocol, 300 µm thick brain slices were stained using the FD Rapid Golgi Stain kit (FD NeuroTechnologies, INC, Columbia, MD, USA). Briefly, slices were impregnated in a mixture of equal volumes of solutions A and B, which were changed after 24 h and stored for seven days in the dark. The brains were transferred to solution C for four days, with a solution change in the first 24 h. Then, the slices were developed with solutions D and E for 10 min, washed, stained, and mounted with Entellan (Merck, Darmstadt, Germany). Images were obtained with a wide-field Zeiss Cell Observer (Zeiss, Oberkochen, Germany), using 20×/0.4 (NA 0.4, LD Plan-NEOFLUAR) or 100X (NA 1.4×, Plan-APOCHROMATIC) objectives, and an AxioCam MRM 12-bit CCD camera (1388 × 1040 pixel). The microscope was controlled with the AxioVis40x64 V4.9.1.0 software.

**Sholl analysis and skeleton:** The image contrast was enhanced before drawing by applying to subtract the background with rolling ball radius = 50 pixels using a Fiji Macro. Neurons were drawn from the improved images in NeuTube (build 1.0z.2018.07) and imported to Fiji with the Simple Neurite Tracer (SNT) plugin (v4.1.13) [34,35]. The neurons were skeletonized, and the Sholl analysis was performed using 5 μm spaced rings from the cell body [36]. Skeleton analysis was automatically performed on a 3D skeleton of the neurons through a homemade Fiji macro. The number of junctions, endpoints, and longest path length were compared between conditions.

**Evaluation of Dendritic Spines:** Individual dendrites were manually selected using a home-made Fiji macro, including the following steps: 40 μm length selection, z stack projection, and manually corrected thresholds (NIH, Bethesda, MD, USA, https://imagej.net/ij (accessed 22 march 2025)).

Dendrite images were converted to probability file images using the Ilastik 1.3.3 tool [37] (interactive machine learning for [bio]image analysis) differentiating between signal and background in each data set; the HDF5 probability files were imported into Fiji using the Ilastik plugin and made into binary followed by thinning and then pruning of the objects within MorphoLibJ-plugin (Plugins>MorphoLibJ>Label Images), Skeletonize, and Analyze>Skeleton plugins [38]. Selected parameters of spine morphology (moments of inertia, volume, branching length, and branching number) were analyzed.

The number of spines, spine density along the dendrite, and quantitative morphological descriptors were calculated from the 2D projection of single dendrite stacks using BoneJ2 (release 7.0.14) [39]. The spine shapes were defined using the morphological descriptors as follows: (i) filopodia (F), length ≥ 2 μm; (ii) mushroom (M), area > 1.5 μm and >0.6 μm in width; (iii) stubby (S), <1 μm in length, >5 areas, and <0.5 µm; and (iv) thin (T), <1 μm length and <0.5 in width [40]. Objects with ≤0.014 or >0.2 in the box axis (=box length × box width) were excluded from the analysis since they either were regarded as unrelated to spine morphology or presented less of their volume within the slices selected for segmentation. We could identify between 1 and 3 whole neurons from each brain. Thus, for comparison analysis, we included the total number of neurons found in each experimental group (13 neurons from 6 brains from each group).

**RNA extraction and quantitative real-time reverse transcription-polymerase chain reaction (RT-qPCR):** Samples from the prefrontal, motor, and auditory cortices from male brains (CD, *n* = 5; HFD, *n* = 5; and HFD-FO, *n* = 4) and female brains (CD, *n* = 5; HFD, *n* = 6; and HFD-FO, *n* = 4), were stored in RNAlater^TM^ (stabilization solution) and further homogenized in 500 uL of TRI reagent (T9424, Sigma-Aldrich, St Louis, MO, USA) for total RNA isolation following the manufacturer’s instructions.

Each rat used for this analysis belonged to a different litter. RNA samples were treated with DNase (18068-015, Invitrogen, Waltham, MA, USA), and cDNA was synthesized from 30 ng of total RNA using the High-Capacity RNA-to-cDNA kit (4387406, Applied Biosystems, Warrington, UK). Quantitative real-time PCR for gene analysis was performed on ARIA-MX (Agilent, Santa Clara, CA, USA) with SYBR Green PCR Master Mix (Applied Biosystems). Primer sequences are listed in the Supplementary Table S2. Relative gene expression was calculated using the comparative cycle threshold method, *Ppia* was used as an endogenous control, and mRNA expression was presented as 2^−ΔΔCt^.

**Analysis of fatty acid by gas chromatography:** Samples from prefrontal, motor, and auditory cortices were obtained from 5 brains of CD, 5 of HFD, and 5 of HFD-FO. Each rat used for this analysis was born in a different litter. The extraction of total lipids from tissues was performed according to the method described by Bligh & Dyer; tissues (~150 mg per sample) were homogenized with a chloroform/methanol mixture (2:1 *v*/*v*) in a cold environment [41]. An amount of 0.5 N MgCl_2_ with 0.05% butylated hydroxytoluene (BHT) was added to the homogenized product and centrifuged at 0–4 °C (3500 rpm × 5 min) to collect the chloroform phase. The samples were then dried at room temperature, and fatty acid methyl esters (FAME) were prepared using the technique described by Morrison and Smith [42]. Saponifiable lipids obtained from tissues were prepared with boron trifluoride (20% methanolic solution), followed by treatment with NaOH saturated in methanol (0.5 N). Finally, FAs were extracted and collected for quantification by gas–liquid chromatography (GC) on an Agilent Hewlett-Packard (model 7890B serial CN13523084, Santa Clara, CA, USA) using a capillary column (HP-5 30 m × 320 μm × 0.25 μm, Agilent) and a flame ionization detector. FAs were identified by comparing their retention times and using C23:0 fatty acid as an internal standard (Nu-Chek Prep Inc., Elysian MN, USA). The results were expressed as percent moles of fatty acid.

**Statistical analysis:** The sample size was calculated using the software G-Power 3.1.9.7, considering that FO prevented a change of 50% with a 25% standard deviation in at least one neurodevelopmental reflex. Then, to obtain a significance of 5% with an 80% power, it was necessary to include a minimum of 5 dams per experimental group. All analyses were performed according to the sex of the PND22 offspring. To avoid the litter effect, data on biometric parameters and neurological reflexes in the offspring were expressed as litter averages, whereas morphological analysis, fatty acid profiling, and transcript quantification were performed in one male and one female born from each litter [43]. The data were expressed as mean ± SEM. The normality and homogeneity of variance were checked using the Shapiro–Wilk normality test and the Brown–Forsythe test, respectively. Differences in body weight gain and food intake were calculated by two-way ANOVA, followed by the Tukey test. Comparisons between groups were performed by one-way ANOVA analysis, followed by Tukey’s multiple comparisons when data passed normality and homogeneity of variance tests. Otherwise, Kruskal–Wallis analysis followed by Dunn’s tests was used. All analyses were performed in GraphPad Prism (GraphPad Prism version 10.2.3, San Diego, CA, USA). A *p*-value of less than 0.05 was considered a criterion of significance

## 3. Results

**Offspring’s biometric characteristics:** The HFD group litters gained more weight than the CD group litters from PND16 until weaning at PND22. Indeed, male and female pups were heavier in HFD than CD at PND22 (*p* = 0.009 and 0.015) (Supplementary Figure S2).

Liver weights were lower in HFD and HFD-FO than in the controls in male (*p* = 0.004; *p* = 0.007) and female offspring (*p* = 0.001; *p* = 0.030, respectively). Regarding fat depots, inguinal, retroperitoneal, and mesenteric fat were higher in the HFD group compared with the controls, in both male (*p* = 0.001; *p* = 0.001; *p* = 0.014, respectively) and female offspring (*p* = 0.004; *p* = 0.001; *p* = 0.020, respectively). In addition, male offspring of the HFD-FO group had lower mesenteric fat than CD (*p* = 0.016) (Supplementary Table S3).

- **Maternal FO supplementation counteracted HFD-induced effects on the offspring’s neurological reflexes and dendrite morphogenesis.**

**Figure 1:**
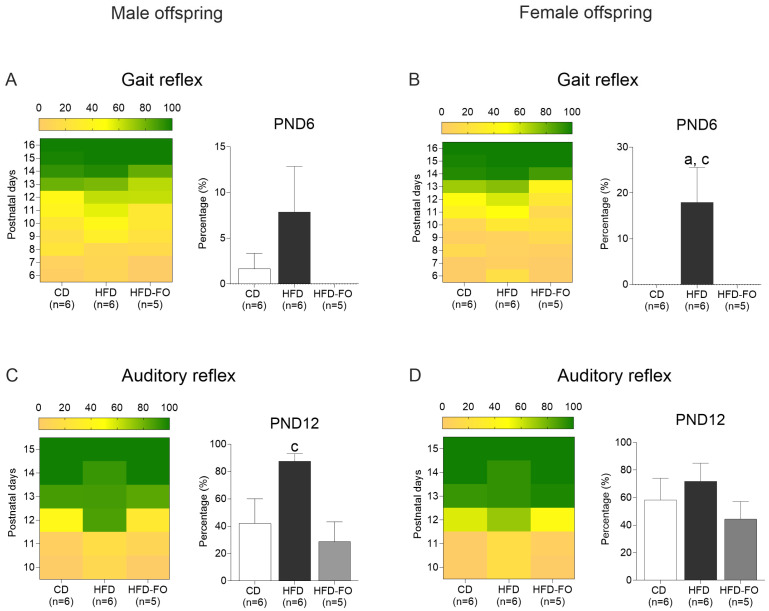
Consolidation of the gait (**A**,**B**) and auditory reflex (**C**,**D**) in male and female litters born to dams fed with control diet (CD), high-fat diet (HFD), and HFD enriched with fish oil (HFD-FO). Heat maps show the mean percentage of animals in each litter that achieved the reflex. Graph bars show the PND when it was expected that animals acquired the reflexes. Litters per group were CD (*n* = 6 per sex), HFD (*n* = 6 per sex), and HFD-FO (*n* = 5 per sex). Data are expressed as mean ± SEM. Differences were calculated by one-way ANOVA, followed by Tukey’s post-test or Kruskal–Wallis analysis, followed by Dunn’s tests. ^a^ *p* < 0.05 between CD vs. HFD, and ^c^ *p* < 0.05 between HFD vs. HFD-FO.

**Neurological reflexes:** To assess the maturation of the nervous system, we evaluated the development of neurological reflexes, defined as involuntary and repetitive movements that appeared according to the maturation of neuronal processes of cortical networks (1). The gait reflex at PND6 was achieved in more females of the HFD group compared with the CD group (*p* = 0.027) and the HFD-FO group (*p* = 0.035). The auditory reflex was achieved at PND12 in a greater number of males of the HFD group compared with the HFD-FO (*p* = 0.019) and was near the level of significance compared with CD (*p =* 0.074) (Figure 1). No differences between groups were shown in the lower grip, upper grip, turning, edge avoidance, posture, eye-opening, and posture correction reflexes; see Supplementary Figure S3.

**Figure 2:**
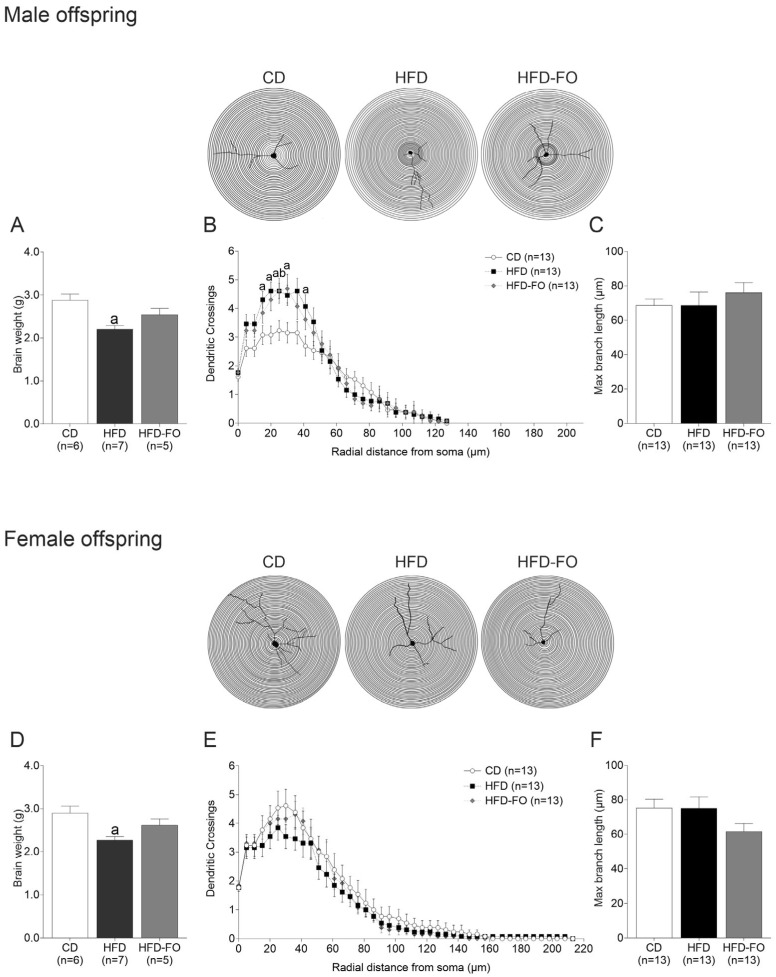
Morphometric analysis of cerebral cortex neurons at PND22 in male and female litters born to dams fed with control diet (CD), high-fat diet (HFD), and HFD enriched with fish oil (HFD-FO). Brain weight in male and female offspring (**A**,**D**). Sholl analysis of cerebral cortex neurons. The schematic representation of morphometric Sholl analysis is shown in the circles (**B**,**E**). The number of intersections between dendrites and concentric spheres centered on the soma was determined at various distances from the soma (5 μm increments). Maximum branch length neurons per group for CD (*n* = 13 per sex), HFD (*n* = 13 per sex), and HFD-FO (*n* = 13 per sex) (**C**,**F**). Data are expressed as mean ± SEM. Differences were calculated by one-way ANOVA followed by Tukey’s post-test or Kruskal–Wallis analysis followed by Dunn’s tests. ^a^ *p* < 0.05 between CD vs. HFD, and ^b^ *p* < 0.05 between CD vs. HFD-FO. Neuron depictions were processed in Procreate software version 5.3.10, 2024.

**Brain weight in the offspring:** The brain weight was lower in both males (*p* = 0.003) and females (*p* = 0.008) of the HFD group compared with CD (Figure 2A,D).

**Quantification of total cell and neuronal density:** A cell and neuronal density analysis was performed to assess if maternal diet interventions altered cellularity in the cerebral cortex’s medial, dorsal, and dorsolateral areas. No differences between groups were observed in the total cell density of both male and female offspring (Supplementary Figure S4). Similar results were observed for neuronal density, but in the dorsolateral area of females, the neuron number was near the significance level in HFD-FO compared with HFD (*p* = 0.054) (Supplementary Figure S5).

**Dendritic arborization:** Sholl analysis was carried out to evaluate if maternal diet affected the number of dendrites in the cerebral cortex of the offspring. In males, a higher number of dendrite branches between 15 to 30 µm (*p* < 0.05 for all comparisons) and 40 µm (*p* = 0.039) were observed in the HFD group compared with the controls (Supplementary Table S4). Moreover, regarding branches of 25 µm, the HFD-FO group showed more branches than the controls (*p* = 0.049) (Figure 2B). In females, no differences were observed (Figure 2E). In turn, the maximum dendrite length was comparable between groups in both sexes (Figure 2C,F).

**Figure 3:**
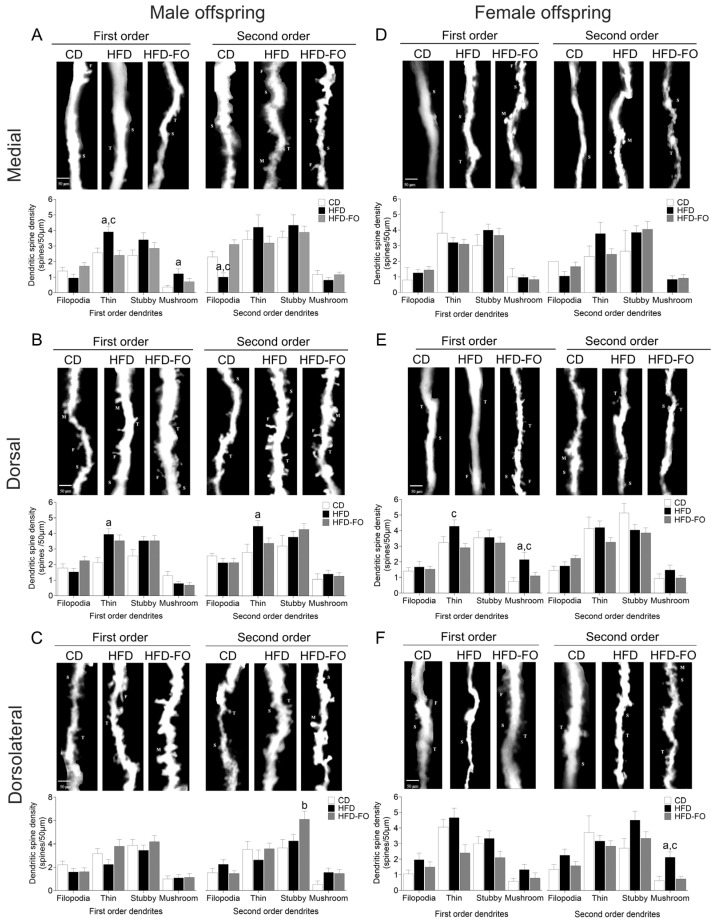
Numbers of each spine type in first- and second-order dendrites of pyramidal neurons in medial, dorsal, and dorsolateral at PND22 in males (**A**–**C**) and females (**D**–F) in offspring born to dams fed with control diet (CD), high-fat diet (HFD), and HFD enriched with fish oil (HFD-FO). Images show the representative morphology of first- and second-order dendrites in each experimental group (F, filopodia; T, thin; S, stubby; and M, mushroom). Graphs show the number of each type of spine in length of 50 µm from CD (*n* = 13 per sex), HFD (*n* = 13 per sex), and HFD-FO (*n* = 13 per sex). Data are expressed as mean ± SEM. Differences were calculated by one-way ANOVA, followed by Tukey’s post-test or Kruskal–Wallis analysis, followed by Dunn’s tests. ^a^ *p* < 0.05 between CD vs. HFD, ^b^ *p* < 0.05 between CD vs. HFD-FO, and ^c^ *p* < 0.05 between HFD vs. HFD-FO.

**Spine subtype analysis:** In the medial zone of the cerebral cortex of male offspring, the density of thin spines in first-order dendrites was higher in the HFD group compared with controls and HFD-FO (*p* = 0.012 and *p* = 0.005, respectively), whereas the mushroom spine type was more predominant in HFD than in CD (*p* = 0.020). In turn, filopodia spines were lower in the HFD group compared with the CD and HFD-FO groups (*p* = 0.043 and *p* < 0.001) in second-order dendrites (Figure 3A). Moreover, the diameter of second-order dendrites was smaller in HFD compared with CD and HFD-FO (*p* = 0.003 and *p* < 0.001).

In the dorsal zone, the first- and second-order dendrites showed more thin-type spines in the HFD group than in the CD group (*p* = 0.002 and *p* = 0.022, respectively) (Figure 3B). The diameter of first-order dendrites in HFD-FO was higher than in the control group (*p* = 0.030). Finally, there were more stubby spines in the dorsolateral zone in the HFD-FO group compared with the CD group (*p* = 0.033) (Figure 3C).

In female offspring, the number of thin spines was higher in first-order dendrites of the dorsal zone of HFD compared with HFD-FO (*p* = 0.013) (Figure 3E). Similarly, mushroom spines were more abundant in the HFD group than in the control and HFD-FO groups in first-order dendrites of the dorsal zone (*p* = 0.014 and *p* = 0.037, respectively) and second-order dendrites of the dorsolateral zone (*p* = 0.005 and *p* = 0.002, respectively) (Figure 3E,F). In the medial zone, no differences in the numbers of different spine types were observed between groups.

- **Maternal FO supplementation restored the HFD-induced effects on the fatty acid profile in the cerebral cortex of the offspring.**

As fatty acids, mainly *n*-3 and *n*-6 polyunsaturated fatty acids, are important regulators of dendrite branching and spine density, and their levels in the brain are influenced by dietary interventions [15], we determined the FA composition in the prefrontal, motor, and auditory cortices of the offspring.

**Figure 4:**
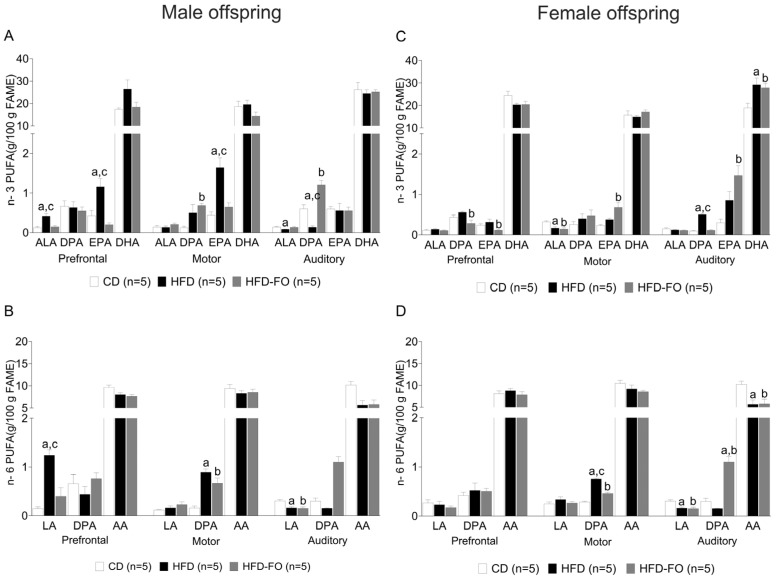
Composition of *n*-3 and *n*-6 polyunsaturated fatty acid (PUFA) in the prefrontal, motor, and auditory areas of the cerebral cortex at PND22 of male (**A**,**B**) and female (**C**,**D**) offspring born to dams fed with the control diet (CD), a high-fat diet (HFD), and an HFD enriched with fish oil (HFD-FO). CD (*n* = 5 mice per sex), HFD (*n* = 5 mice per sex), and HFD-FO (*n* = 5 mice per sex). Data are expressed as mean ± SEM. Differences were calculated by one-way ANOVA followed by Tukey’s post-test or Kruskal–Wallis analysis followed by Dunn’s tests. ^a^ *p* < 0.05 between CD vs. HFD, and ^b^ *p* < 0.05 between CD vs. HFD-FO, and ^c^ *p* < 0.05 between HFD vs. HFD-FO.

In the prefrontal cortex of male offspring, a higher concentration of the *n*-3 PUFAs α-linolenic acid (ALA) and eicosapentaenoic acid (EPA) was observed in the HFD group compared with the CD (*p* = 0.001 and *p* = 0.011, respectively) and HFD-FO (*p* = 0.003 and *p* = 0.001, respectively) groups. A similar situation was found in the motor cortex, where the HFD group presented elevated levels of EPA compared with CD and HFD-FO (*p* = 0.005; *p* = 0.002, respectively). In addition, it was observed that the HFD-FO group presented an increase in docosapentaenoic acid of the *n*-3 series (DPA *n*-3) compared with CD (*p* = 0.018). Finally, in the auditory cortex, the HFD group presented a lower level of ALA than CD (*p* = 0.032), and DPA *n*-3 in the HFD group was lower than in CD and HFD-FO (*p* = 0.006; *p* = 0.009, respectively) but was higher in HFD-FO than in CD (*p* < 0.001) (Figure 4A).

A similar result was obtained for *n*-6 PUFAs in the prefrontal cortex; linoleic acid (LA) was higher in HFD compared with CD and HFD-FO (*p* < 0.001, *p* < 0.001). In the motor cortex, DPA of the *n*-6 series (DPA *n*-6) was lower in CD compared with HFD (*p* < 0.001) and HFD-FO (*p* = 0.001). Finally, in the auditory cortex, CD showed higher LA content compared with HFD and HFD-FO (*p* < 0.001 and *p* < 0.001) (Figure 4B).

The composition of saturated fatty acids (SFAs) and monounsaturated fatty acids (MUFAs) was characterized by higher levels of C15:0 (pentadecanoic acid) in the prefrontal cortex (*p* = 0.022) and lower levels in the motor and auditory cortex (*p* = 0.008 and *p* = 0.013, respectively) in the HFD group compared with the CD group. Moreover, the levels of C15:0 were also lower in HFD-FO than in CD (*p* = 0.038). C14:1 (myristoleic acid) levels were higher in the HFD group compared with the CD group in the prefrontal and auditory cortices (*p* = 0.030 and *p* = 0.007, respectively). In turn, C22:1 *n*-9 (erucic acid) was higher in HFD-FO compared with CD in the motor cortex (Supplementary Tables S5–S7).

In the female offspring, DPA *n*-3 and EPA content in the prefrontal cortex was higher in HFD than in HFD-FO (*p* = 0.016; *p* = 0.030). In the motor cortex, ALA was higher in CD compared with HFD and HFD-FO (*p* = 0.012 and *p* = 0.004, respectively). In addition, EPA increased in HFD-FO compared with CD (*p* = 0.003). On the other hand, HFD presented more DPA *n*-3 than CD and HFD-FO (*p* < 0.001), whereas in the auditory cortex, DPA *n*-3 was higher in HFD compared with CD and HFD-FO (*p* < 0.001 and *p* < 0.001, respectively). Finally, DHA was lower in CD than in HFD and HFD-FO (*p* = 0.023; *p* = 0.048), while EPA was higher in HFD-FO than in CD (*p* = 0.003) (Figure 4C).

Regarding *n*-6, DPA was higher in HFD compared with CD and HFD-FO (*p* < 0.001 and *p* < 0.001) in the motor cortex; also, HFD-FO was higher than CD (*p* < 0.001). In the auditory cortex, LA and arachidonic acid (AA) were higher in CD than HFD (*p* = 0.005; *p* = 0.014) and HFD-FO (*p* = 0.002; *p* = 0.017). Finally, DPA *n*-6 was higher in HFD-FO compared with HFD and CD (*p* < 0.001) (Figure 4D).

The level of C15:0 was lower in HFD compared with CD and HFD-FO in the prefrontal cortex (*p* = 0.008 and *p* = 0.013, respectively) and motor cortex (*p* = <0.001 and *p* = 0.004, respectively). In the same way, lower levels of C15:0 were found in HFD compared with CD in the auditory cortex (*p* = 0.002). In turn, lower levels of C16:1 (palmitoleic acid) were observed in HFD compared with CD (*p =* 0.031) in the motor cortex. In the auditory cortex, C14:1 was higher (*p* <0.001 and *p* <0.001, respectively), and C18:1 *n*-9 (oleic acid) and total MUFAs were lower in HFD and HFD-FO compared with CD (*p* = 0.020 and *p* = 0.027, respectively). On the other hand, C16:1 was lower in HFD and HFD-FO compared with CD (*p* = 0.005 and *p* = 0.039, respectively) (Supplementary Tables S5–S7).

We also evaluated the FA profile in the liver because brain accretion depends on nutrition and metabolism. In males, HFD showed lower levels of C14:0 (*p* < 0.001), C16:1 (*p* < 0.001), DHA, and total *n*-3 PUFAs (*p* < 0.001 and *p =* 0.001) than CD and HFD-FO (*p* = 0.019; *p* = 0.049; *p* <0.001; *p* <0.001, respectively), and C14:1 was lower in HFD compared with CD (*p* = 0.007), whereas C15:0 and C16:0 were higher in CD compared with HFD (*p* < 0.001 and *p* < 0.001) and HFD-FO (*p* = 0.005 and *p* < 0.001). AA, total MUFAs, PUFAs, and PUFAs *n*-6 were higher in HFD-FO than CD and HFD (*p* = 0.012; *p* = 0.011; *p* = 0.001 and *p* = 0.005, respectively). On the other hand, C18:0 and the *n*-6:*n*-3 ratio were higher in HFD compared with CD (*p* = 0.001 and *p* < 0.001) and HFD-FO (*p* = 0.002 and *p* = 0.006, respectively) (Supplementary Table S8).

In females, C14:0, C15:0, C16:0, and LA were higher in CD than HFD (*p* = 0.006, *p* < 0.001, *p* < 0.001, and *p =* 0.028, respectively) and HFD-FO (*p* = 0.034, *p* < 0.001, *p* = 0.003, and *p =* 0.006, respectively). For its part, C20:0, C18:1 *n*-9, AA, DPA *n*-6, DHA, total MUFAs, PUFAs, and *n*-3 PUFAs were lower in CD compared with HFD-FO (*p* <0.001, *p* <0.001, *p* <0.001, *p* <0.001, *p =* 0.049, *p* <0.001, *p* <0.001, and *p =* 0.021, respectively) and HFD (*p* = 0.001, *p* < 0.001, *p =* 0.014, *p* < 0.001, *p* <0.001, *p* <0.001, *p* <0.001, and *p* < 0.001, respectively). The levels of *n*-6 PUFAs were higher in HFD compared with CD (*p* = 0.017) but lower compared with HFD-FO (*p* = 0.002), whereas C18:0 and the n6:*n*-3 ratio were higher in HFD compared with CD (*p* = 0.001). In turn, C20:3 *n*-3 was higher in HFD (*p* <0.001) and HFD-FO (*p* <0.001) compared with CD (Supplementary Table S8).

- **Maternal FO supplementation increased transcript levels of *Bdnf* in the cerebral cortex of male offspring.**

Based on our previous results, we performed transcript quantification on the same areas of the cerebral cortex evaluated for FA analysis of the following genes: *Snap25* and *Bdnf* encoding for the proteins associated with synaptic function, SNAP-25 and BDNF; *Mfsd2a* encoding for MFSD2a, a fatty acid transporter of DHA; *Nfkb1* encoding for the subunit p50 of transcription factor NF-κB, which regulates the transcription of a variety of inflammatory factors; and *Il1b* encoding for IL-1β, a proinflammatory cytokine.

**Figure 5:**
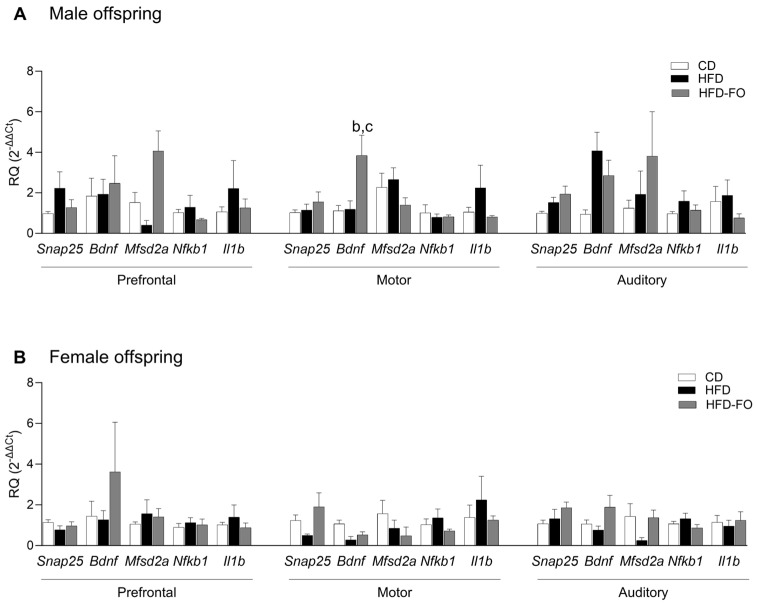
Relative transcript quantification (RQ) of *Snap25*, *Bdnf*, *Mfsd2a*, *Nfkb1*, and *Il1b* genes in the prefrontal, motor, and auditory areas from the cerebral cortex at PND22 of male (**A**) and female (**B**) offspring born to dams fed with control diet (CD), high-fat diet (HFD), and HFD enriched with fish oil (HFD-FO). CD (*n* = 3–5 males and *n* = 4–5 females), HFD (*n* = 4–5 males and *n* = 5–6 females), and HFD-FO (*n* = 4 males and *n* = 4 females). RQ was calculated using *Ppia* as a housekeeping gene. Data are expressed as mean ± SEM. Differences were calculated by one-way ANOVA followed by Tukey’s post-test or Kruskal–Wallis analysis followed by Dunn’s tests. ^b^ *p* < 0.05 between CD vs. HFD-FO, and ^c^ *p* < 0.05 between HFD vs. HFD-FO.

In male offspring, the levels of the *Bdnf* transcript were higher in HFD-FO compared with CD and HFD in the motor cortex (*p* = 0.019 and *p* = 0.016, respectively); moreover, in the auditory cortex, *Bdnf* tended to be higher in HFD compared with CD (*p* = 0.067) (Figure 5A). In females, the levels of *Snap25* and *Bdnf* were close to significance between HFD-FO and HFD in the motor and auditory cortices (*p* = 0.088 and *p* = 0.072, respectively) (Figure 5B). No differences between groups were found in the transcripts of *Mfsd2a, Nfkb1,* and *Il1b* (Figure 5A,B).

## 4. Discussion

FO supplementation from pregnancy to the end of lactation counteracted the accelerated gain of auditory reflexes in males and motor reflexes in females induced by HFD. Moreover, it partially attenuated the changes provoked by HFD on the morphology of dendritic spines in the cerebral cortex, regulating the number of thin and mushroom spine types in the offspring of both sexes. These morphologic and functional features were also accompanied by gene expression changes in males, with an upregulated *Bdnf* transcript level in the motor cortex and a tendency to increase the mRNA level of *Snap25* and *Bdnf* in the motor and auditory cortices in females. In addition, it increased the DHA content in the female offspring’s auditory cortex. Thus, we found that FO supplementation was associated with an increase in the expression of genes linked with the regulation of synaptic transmission and plasticity that may have a direct effect on the morphological and functional changes induced by HFD in the auditory and motor cortices, recovering the function of these structures.

In our model, which included dietary interventions during pre-pregnancy, pregnancy, and lactation, HFD induced an early gait acquisition in female offspring and auditory startle reflexes in male offspring. Interestingly, FO-supplemented rats and mice got closer to the timing of acquisition of these neurological reflexes to the control diet group, probably due to its impact on neurogenesis, synaptogenesis, migration, or axonal myelination [44,45]. The gait reflex involves locomotion and engages various brain areas, such as the motor cortex, basal ganglia, midbrain, hindbrain, and spinal cord [46]. In turn, auditory startle involves the synaptic connections of giant neurons in the brainstem; however, circuits in layers 3 to 5 of the auditory cortex also modulate this reflex [47]. The growth and complexity of dendritic arbors are central to developing neural circuits, which increase their surface area and connectivity, and impairments in dendrite morphogenesis have been associated with neurodevelopmental disorders [48]. Similar to our findings of increased dendrite branching in male offspring and a predominance of thin and mushroom spines in dendrites of both sexes induced by HFD, rats exposed to valproic acid (VPA), a rodent model of autism spectrum disorder (ASD), showed an exacerbated dendrite branching with an increased proportion of thin and mushroom-type spines that had a reduction in head diameter resulting in an unstable synapse in the cortex and hippocampus [49,50]. Moreover, there is increased locomotor activity at an early age and earlier acquisition of the auditory startle reflex in male pups [51,52]. In this regard, one theory about the origin of autism is based on increased short-range dendritic arborization, which may result in local over-connectivity but disrupted long-term connectivity [53]. In addition, studies that have included treatment with HFD during pregnancy and lactation have led to autistic-like behaviors with increased hyperactivity during adolescence of the offspring [54,55]. Then, the similarities of our observations in cortical areas of the brain and neurodevelopmental reflexes with previous rodent models of ASD allow us to suggest that HFD leads to neurological abnormalities that resemble, at least in part, those observed in this condition. To our knowledge, not many studies have analyzed the effects of maternal HFD on the brain cortex of offspring. However, it has been observed that males exposed to maternal HFD at PND28 show an altered expression of ASD-related genes encoding proteins involved in three pathways, synaptic function, chromatin remodeling, and transcription regulation [56], which supports our observations.

Therefore, the early acquisition of neurological reflexes induced by HFD does not imply a gain of function, because the processes involved in brain development are finely time-tuned, starting before and continuing after birth and lactation, when the processes of myelination and synaptogenesis are continuously taking place [57]. In this regard, our data suggest that *n*-3 PUFAs contained in FO could not modify the pattern of dendrite branching but attenuated the effects on dendrite spines, reducing the predominance of thin and mushroom spines, suggesting that *n*-3 PUFAs can modulate differential pathways that control dendrite outgrowth and spine turnover. Spines can be classified according to their shape into four types (mushroom, stubby, thin, and filopodia), which are closely linked with their function. Therefore, changes in the density and types of spines must be considered good markers of dysfunction at the cellular or circuit level [58]. Thin spines are considered immature with a high turnover rate, forming weak or silent synapses, but can acquire a full functional synapse if they progress into stable mushroom spines [59]. In this regard, FO supplementation regulates the dynamics of spine maturation because it normalizes the number of filopodia, thin, and mushroom types. Interestingly, filopodia are known to serve as precursors for forming new spines, suggesting that FO could promote synaptogenesis processes [60]. Multiple signals regulate spine density and morphogenesis, including neurotransmitters, proteins of the extracellular matrix, contact-mediated ligands, and neurotrophins [61]. Among these signals, we observed that the gene expression of brain-derived neurotrophic factor *(Bdnf*), which acts mainly in the regulation of spine remodeling, was increased in the motor area of males and tended to be higher in the auditory area of females of FO compared with HFD. It has been shown that *n*-3 PUFAs promote BDNF expression by activating G-protein coupled receptor 40 (GPR40) and p38 mitogen-activated protein kinases [62,63]. In turn, BDNF, through protein kinase B (Akt) and Ca2+/calmodulin-dependent protein kinase (CaMKII), can stimulate the growth of dendrites and increase the spine density of cortical pyramidal neurons in organotypic brain slices [64,65]. On the other hand, *Snap25*, which tended to be higher in FO in the motor area of female offspring, plays an essential role in neurotransmitter release, neurite extension, and sprouting [66,67]. Therefore, FO supplementation promotes neurotrophic factors that regulate the effects of HFD on the brain cortex of the offspring. The positive effects of *n*-3 PUFA, mainly DHA, seem not to be restricted to the brain cortex, as it has been observed that it accelerates neurite outgrowth and synaptogenesis in the hippocampus of developing animals and adults, suggesting a potential beneficial effect on memory and learning [24,68].

Sex affects neuron morphology, modifying dendrites’ architecture and spine density, with CA1 and CA3 hippocampal neurons exhibiting a higher branching of basal dendrites in adolescent and young males compared with female rats [69]. Moreover, different susceptibilities of male and female offspring to maternal dietary interventions have been described, with the male fetal brain being more disposed to change its transcriptome [70]. Therefore, it is unsurprising that in our study, males are more prone to respond to HFD and FO supplementation.

There is a high risk of ASD in children of women who were obese during pregnancy [71]; our results may have a clinical translation in this setting. Studies in pregnant women show that the intake of *n*-3 PUFAs during the second trimester of gestation reduces the risk of ASD in children, probably due to their biological effects on brain development [72]. In this regard, maternal obesity impairs placental transfer and/or synthesis of PUFA, reducing the bioavailability of *n*-3 and *n*-6 PUFA (DHA and AA) to the offspring during pregnancy and lactation [73]. Therefore, based on the structural effects on the cerebral cortex, our study supports the beneficial effect of *n*-3 PUFAs in maternal obesity.

During pregnancy, *n*-3 and *n*-6 PUFAs are obtained from the maternal diet and supplied to the offspring through transplacental transfer by the uptake and transport across the membrane through the flip-flop mechanism or binding proteins and transporters [73]. In general, there is a selective enrichment of DHA and AA in the fetal compartment, known as biomagnification, with preferential transport to support their rapid accretion in the fetal nervous tissue during the brain growth spurt [74,75].

On the other hand, during lactation, the DHA and AA content of milk depends on the maternal diet. Interestingly, maternal obesity and obesogenic diets reduce both transplacental transfer and milk content of DHA and AA, affecting normal neurological development [76,77,78]. Therefore, the maternal dietary supply of PUFAs is crucial for the offspring’s pre- and post-natal brain development. We found that in the prefrontal cortex of male offspring born to HFD dams, the ALA, EPA, and LA levels were increased compared with those in the CD group. However, in contrast to previous observations in obese adult animals exposed to HFD, DHA and AA levels remained unchanged, suggesting that HFD could exacerbate the uptake of precursors to maintain adequate levels of DHA and AA, indicating that independently of the composition of the diet, the brain can synthesize or transport DHA and AA to preserve their balance, which is essential for neurogenesis, synaptogenesis migration, and axonal myelination [44,45]. According to our results, no differences were observed in the mRNA expression for MFSD2a, suggesting that the transport of DHA in the BBB was not affected by HFD or HFD-FO.

On the other hand, an increase in EPA was observed after FO supplementation, which may reflect compensatory mechanisms in response to dietary imbalance. This suggests that DHA could negatively regulate its own synthesis by inhibiting EPA elongation through the enzyme-specific elongation of very long-chain 2 (ELOVL2) [79]. Finally, the endogenous production of EPA and DHA from their precursors at the brain level must be considered due to their important role in optimal brain maintenance [80,81]. A kinetic study has shown that newly synthesized DHA originating from plasma non-esterified EPA is stored in the liver or packaged into triacylglycerols for secretion into the plasma. However, non-esterified EPA appears to be a significant source of brain DHA turnover, indicating the importance of EPA for maintaining brain DHA levels [82]. In this regard, our results showed that HFD did not modify EPA levels in the offspring’s liver; even more, its levels were higher in the brain cortex of males and to a minor degree in females.

In females, the FA profile was affected mainly in the auditory cortex, showing reduced levels of AA but increased DHA. Therefore, HFD-induced variations in the content and type of FA in the brain cortex are determined by the offspring’s sex. Previous studies support this concept, describing a higher percentage of SFA and lower *n*-6 PUFA levels in males than females [83]. These levels could differ depending on the brain region, indicating that the composition of FAs is fundamental for brain-specific functions [84,85].

Interestingly, FO supplementation normalized the levels of ALA and LA in the brain cortex, suggesting that *n*-3 PUFAs could regulate the uptake of *n*-3 and *n*-6 PUFAs [86]. Moreover, it activates peroxisome proliferator-activated receptors, increasing peroxisomal FAs oxidation [87]. In this regard, ALA and LA are primary substrates for peroxisomal oxidation and energy production in the brain, and they can be increased in conditions of HFD and normalized with FO supplementation. Tight control of LA levels is relevant to avoid oxylipin synthesis, which could be harmful because it makes mitochondria more susceptible to damage in conditions of lower lipid protective antioxidant capacity [88].

In addition to the changes in cortical PUFA composition induced by HFD, we observed a consistent reduction in pentadecanoic acid (C15:0) across all analyzed cortical areas in both male and female offspring. Pentadecanoic acid is an SFA present in maternal milk and originates from propionic acid by the gut microbiome [89]. This metabolite has anti-inflammatory effects and protective roles in metabolic, immune, and neuronal health. Its reduction in the offspring’s brain may reflect an HFD-induced disruption of the maternal gut microbiome and a consequent decrease in beneficial microbial metabolites [90]. Notably, FO supplementation restored C15:0 levels in cortical areas of the offspring, suggesting a potential link between *n*-3 PUFAs and gut-derived neuroactive metabolites. However, as microbiota composition and SCFA concentrations were not assessed in this study, this hypothesis remains speculative and warrants further investigation.

We also examined the expression of genes involved in neuroinflammation (NF-κB and IL-1β), yet no significant changes were detected across experimental groups in contrast. This indicated that in our model, the neuroprotective effects of FO were unlikely to be mediated via canonical inflammatory pathways.

Moreover, we found increased serum levels of myristoleic acid (C14:1) in male offspring’s prefrontal and auditory cortices. In this regard, similar results have been found in subjects with autism, consistent with our data [91].

Although interspecies differences in brain development and metabolism are well recognized, our findings may inform maternal nutritional strategies to prevent neurodevelopmental alterations in humans. In humans, DHA—primarily obtained from fish oil—is a significant brain lipid and plays a critical role in neuronal proliferation, migration, differentiation, and synaptogenesis [92].

A cohort study found that maternal fish oil supplementation before and during pregnancy was associated with improved problem-solving and social skills in children [28]. Likewise, a randomized controlled trial showed that daily supplementation with 1 g of fish oil from the third trimester through early childhood enhanced early language and motor development and reduced emotional and behavioral issues [93].

These findings suggest that maternal fish oil intake could mitigate the adverse effects of a high-fat diet on offspring brain development. However, further studies are needed to establish the relevance of these findings, for example, the long-term impact on cognition, particularly on memory and hippocampal function.

### Limitations and Strengths

Our study has some limitations due to the design that included sampling for morphological and molecular analysis of the cerebral cortex in different areas, which limited the amount of tissue available to perform other analyses, such as the measurement of a panel of pro-inflammatory cytokines, which have been related to the alterations on behavior and neurogenesis induced by HFD and improved with FO administration [54,94,95]. Additionally, further immunohistochemical analyses would demonstrate the distribution of relevant proteins involved in brain plasticity, such as BDNF. On the other hand, the main strength of our study is that it provides information about the effects of dietary interventions considering treatment from pre-pregnancy until lactation, in addition to the evaluation according to offspring sex, which allows us to have a more detailed and specific description of the effects associated with HFD and FO supplementation.

## 5. Conclusions

In conclusion, our study demonstrated that HFD accelerated the acquisition of motor and auditory neurological reflexes and generated changes at the level of neuronal morphology, mainly in male offspring. These findings are probably associated with processes such as synaptogenesis or neurogenesis, which could be affected by maternal HFD. Furthermore, our findings highlight the protective role of dietary supplementation with FO containing EPA and DHA in counteracting some of the offspring’s neurodevelopmental changes due to maternal HFD consumption. These findings may have implications regarding interventions to prevent the deleterious effects of maternal unhealthy dietary patterns on important targets such as the brain cortex and possibly on other brain structures, which need further investigation.

## Figures and Tables

**Table 1:** Diet composition for the three experimental conditions: control (CD), high-fat (HFD), and high-fat supplemented with fish oil (HFD-FO).

Nutritional Composition	CD (D12450H)	HFD (D12451)	HFD-FO (D21030416)
Calories per gram	3.85	4.73	4.73
Protein (%)	20	20	20
Carbohydrates (%)	70	35	35
Lipids (%)	10	45	45
Saturated fatty acids (%)	23.50	31.59	29.27
Stearic acid (g)	3.08	19.78	11.98
Palmitic acid (g)	6.45	36.85	32.46
Monounsaturated fatty acids (%)	29.69	35.51	29.23
Oleic acid (g)	12.32	64.10	41.41
Polyunsaturated fatty acids (%)	46.81	32.91	41.50
Linoleic acid (g)	17.82	56.24	34.30
Arachidonic acid (g)	0.06	0.50	2.27
Linolenic fatty acid (g)	2.12	4.21	4.38
Eicosapentaenoic acid (g)	0.00	0.00	13.85
Docosahexaenoic acid (g)	0.00	0.00	10.00
Omega-6	17.90	56.97	37.27
Omega-3	2.13	4.36	34.04
Ratio omega-6/omega-3	8.39	13.07	1.09

## Data Availability

The original contributions presented in this study are included in the article/Supplementary Materials. Further inquiries can be directed to the corresponding authors.

## Supplementary Materials

The following supporting information can be downloaded at https://www.mdpi.com/article/10.3390/nu17101741/s1: Figure S1. Experimental design. Figure S2. Weight gain in litters from PND3 until weaning (A), and body weight at PND22 in males (B) and females (C) offspring born to dams fed with control diet (CD), high-fat diet (HFD) and HFD enriched with fish oil (HFD-FO). Figure S3. Neurological reflex development of front grip, rear grip, turn, edge avoidance, stance, eye-opening, and stance correction in males and female’s offspring born to dams fed with control diet (CD), high-fat diet (HFD) and HFD enriched with fish oil (HFD-FO). Figure S4. Total cell quantification in medial, dorsal, and dorsolateral areas of brain cortex of male (A–C) and female (D–F) offsprings born to dams fed with control diet (CD), high-fat diet (HFD) and HFD enriched with fish oil (HFD-FO). Figure S5. Neuron density in medial, dorsal, and dorsolateral areas of the brain cortex of male (A–C) and female (D–F) offspring’s born to dams fed with control diet (CD), high-fat diet (HFD) and HFD enriched with fish oil (HFD-FO). Table S1. Composition of fish oil (Menhaden fish oil) incorporated in the high-fat diet. Table S2. Gene primers analyzed in the cerebral cortex of rat pups. Table S3. Biometric parameters in PND22 males and females offspring. Table S4. Intersections of neuronal arborization in PND22 male offspring. Table S5. Determination of fatty acids by gas chromatography in prefrontal cerebral cortex in male and female offspring at PND22. Table S6. Determination of fatty acids by gas chromatography in motor cerebral cortex in male and female offspring at PND22. Table S7. Determination of fatty acids by gas chromatography in auditory cerebral cortex in male and female offspring at PND22. Table S8. Determination of fatty acids by gas chromatography in liver in male and female offspring at PND22.

## Abbreviations

The following abbreviations are used in this manuscript:

AA	Arachidonic acid
ALA	α-linolenic acid
ANOVA	Analysis of variance
ASD	Autism spectrum disorders
BBB	Blood–brain barrier
BDNF	Brain-derived neurotrophic factor
BHT	Butylated hydroxytoluene
CaMKII	Ca^2+^/calmodulin-dependent protein kinase
CD	Control diet
DHA	Docosahexaenoic acid
DPA *n*-3	Docosapentaenoic acid *n*-3 series
DPA *n*-6	Docosapentaenoic acid *n*-6 series
ELOVL2	Enzyme-specific elongation of very long chain 2
EPA	Eicosapentaenoic acid
FAME	Fatty acid methyl esters
FFA	Free fatty acid
GABA	Gamma-aminobutyric acid
GC	Gas chromatography
GPR40	G-protein coupled receptor 40
HFD	High-fat diet
HFD-FO	High-fat diet—fish oil
IL-1β	Interleukin-1β
LA	Linoleic acid
MFSD2a	Major facilitator superfamily domain-containing protein 2
mRNA	Messenger ribonucleic acid
MUFA	Monounsaturated fatty acid
NF-κB	Nuclear factor kappa-light-chain enhancer of activated B cells
PBS	Phosphate buffer saline
PND	Postnatal day
PUFA	Polyunsaturated fatty acid
RT-qPCR	Quantitative reverse transcription polymerase chain reaction
SCFA	Short-chain fatty acids
SFA	Saturated fatty acid
SNAP25	Synaptosome-associated protein 25
VPA	Valproic acid
