# Supplementary material for: Fish Oil Supplementation Attenuates Offspring’s Neurodevelopmental Changes Induced by a Maternal High-Fat Diet in a Rat Model
Source: Nutrients. 2025 May 21;17(10):1741. doi: 10.3390/nu17101741 (PMC12113741; doi:10.3390/nu17101741)

Figure S1. Experimental design. Six-week-old female Sprague Dawley rats were fed a control diet (CD) and a high-fat diet (HFD) for four weeks. Then, after mating, they were subdivided into three groups: CD, HFD, and HFD-FO. They were kept until postnatal day (PND) 22, neurological reflex development was performed. After euthanasia, brains were extracted for morphological analysis and transcript quantification.

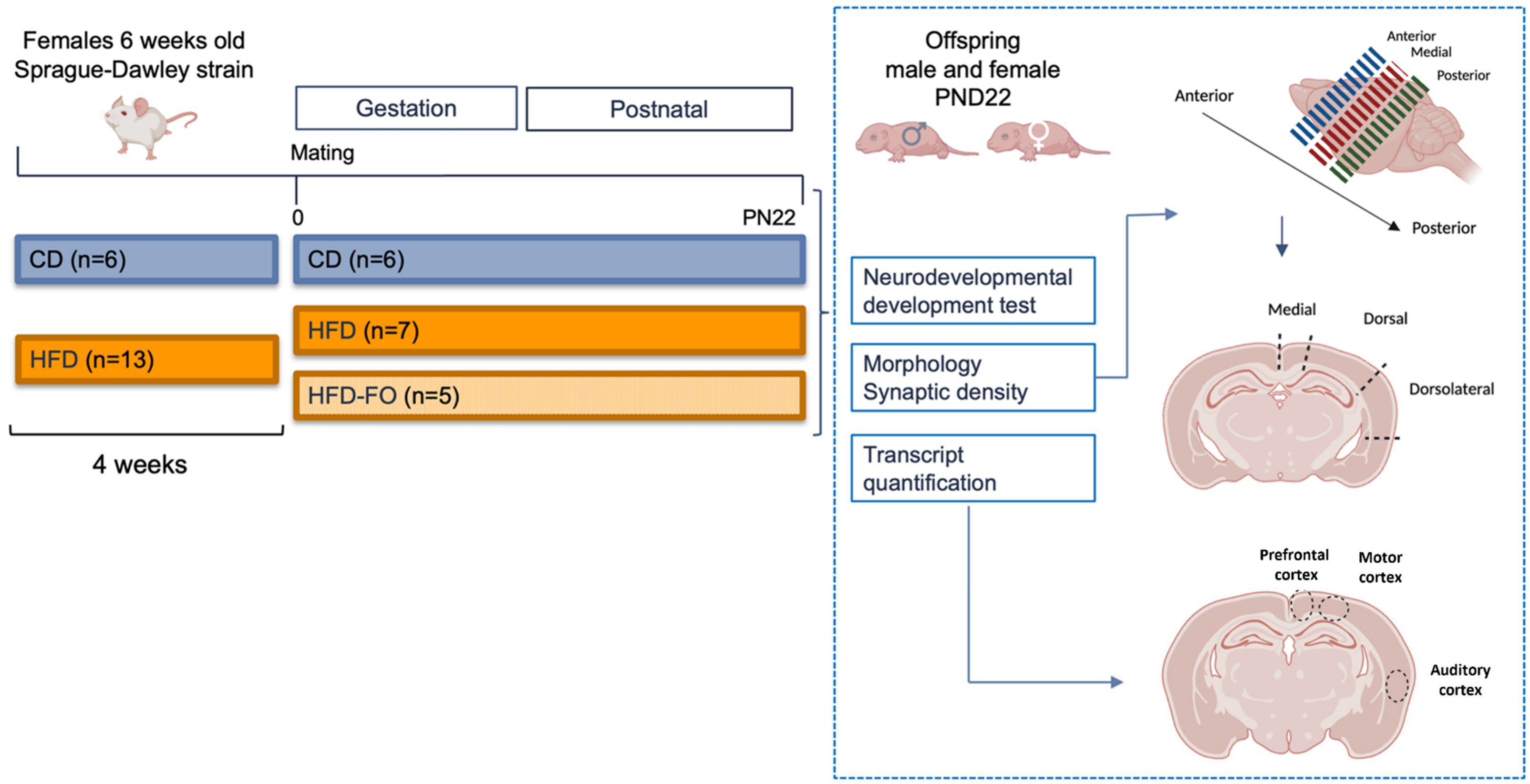

Figure S2. Weight gain in litters from PND3 until weaning (A), and body weight at PND22 in males (B) and females (C) offspring born to dams fed with control diet (CD), high-fat diet (HFD) and HFD enriched with fish oil (HFD-FO). CD (n = 6 mice per sex), HFD (n = 7 mice per sex) and HFD-FO (n = 5 mice per sex). Data are expressed as mean ± SEM. Differences were calculated by ANOVA two-way followed by Tukey's post-test or Kruskal-Wallis analysis followed by Dunn's tests. [a]$p < 0.05$ between CD vs HFD, and [c]$p < 0.05$ between HFD vs HFD-FO.

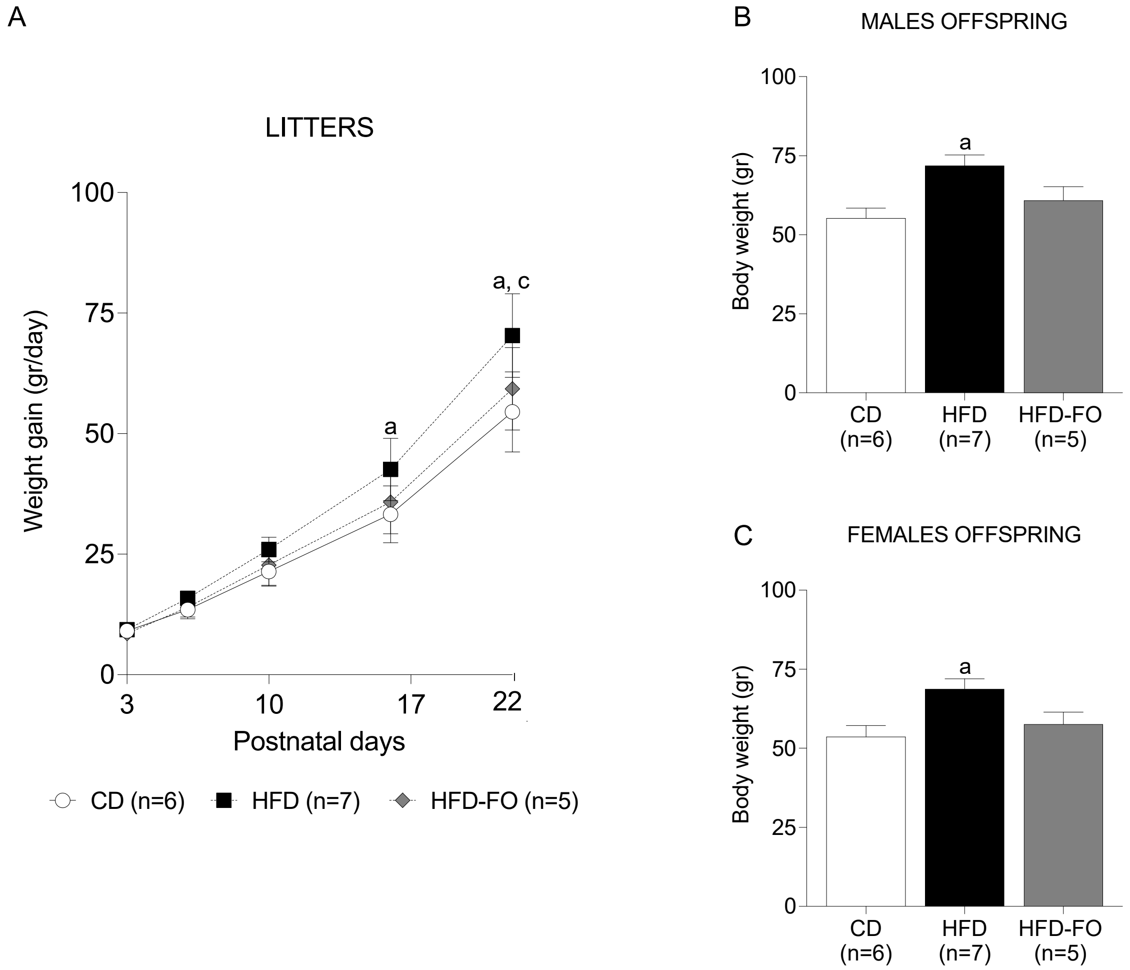

Figure S3. Neurological reflex development of front grip, rear grip, turn, edge avoidance, stance, eye-opening, and stance correction in males and female's offspring born to dams fed with control diet (CD), high-fat diet (HFD) and HFD enriched with fish oil (HFD-FO). CD (n = 6 mice per sex), HFD (n = 6 mice per sex) and HFD-FO (n = 5 mice per sex). Heat maps show the mean percentage of animals in each litter that achieved the reflex. Heat maps show the mean percentage of animals in each litter that achieved the reflex. Differences were calculated by one–way ANOVA followed by Tukey's post-test or Kruskal-Wallis analysis followed by Dunn's tests.

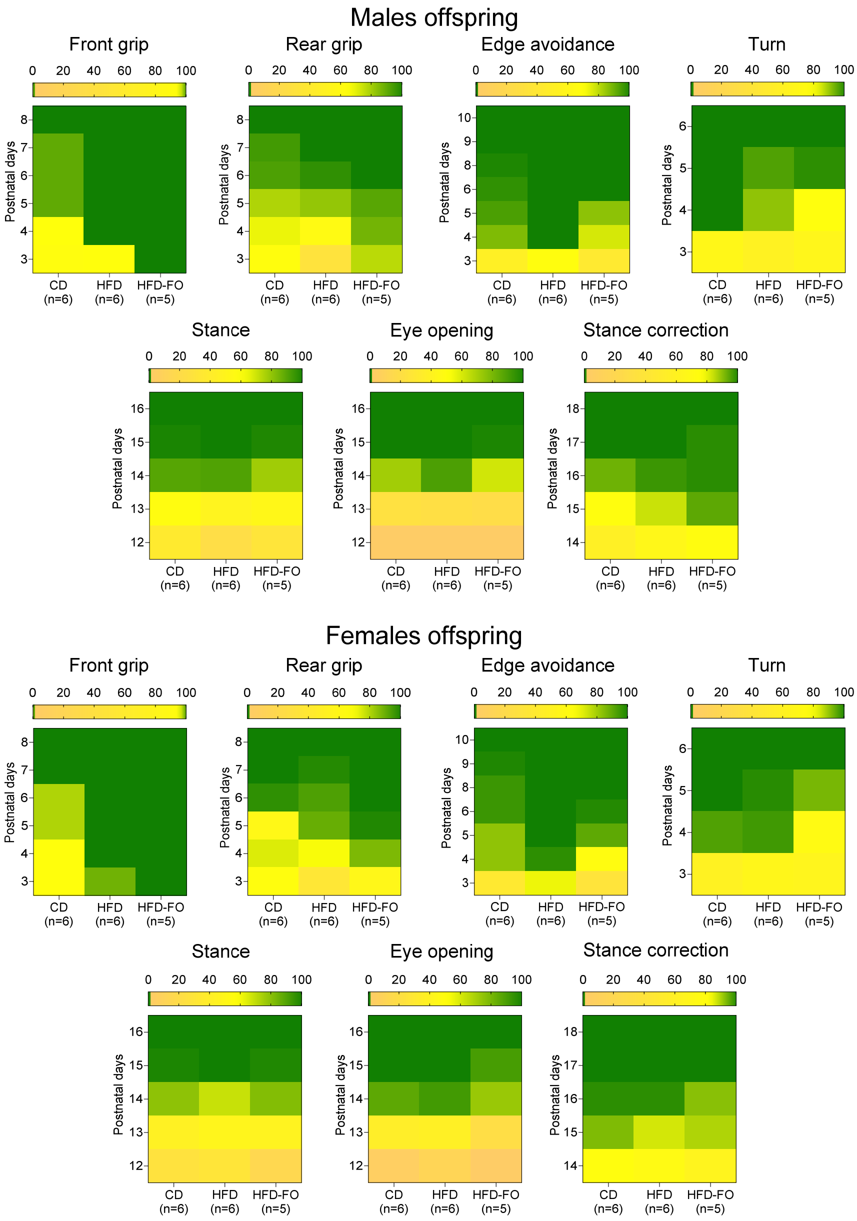

Figure S4. Total cell quantification in medial, dorsal, and dorsolateral areas of brain cortex of male (A–C) and female (D–F) offsprings born to dams fed with control diet (CD), high-fat diet (HFD) and HFD enriched with fish oil (HFD-FO). CD (n = 6 mice per sex), HFD (n = 7 mice per sex) and HFD-FO (n = 5 mice per sex). Box shows representative images of each experimental group in males and females (G). Data are expressed as mean ± SEM. Differences were calculated by one–way ANOVA followed by Tukey's post-test or Kruskal-Wallis analysis followed by Dunn’s tests.

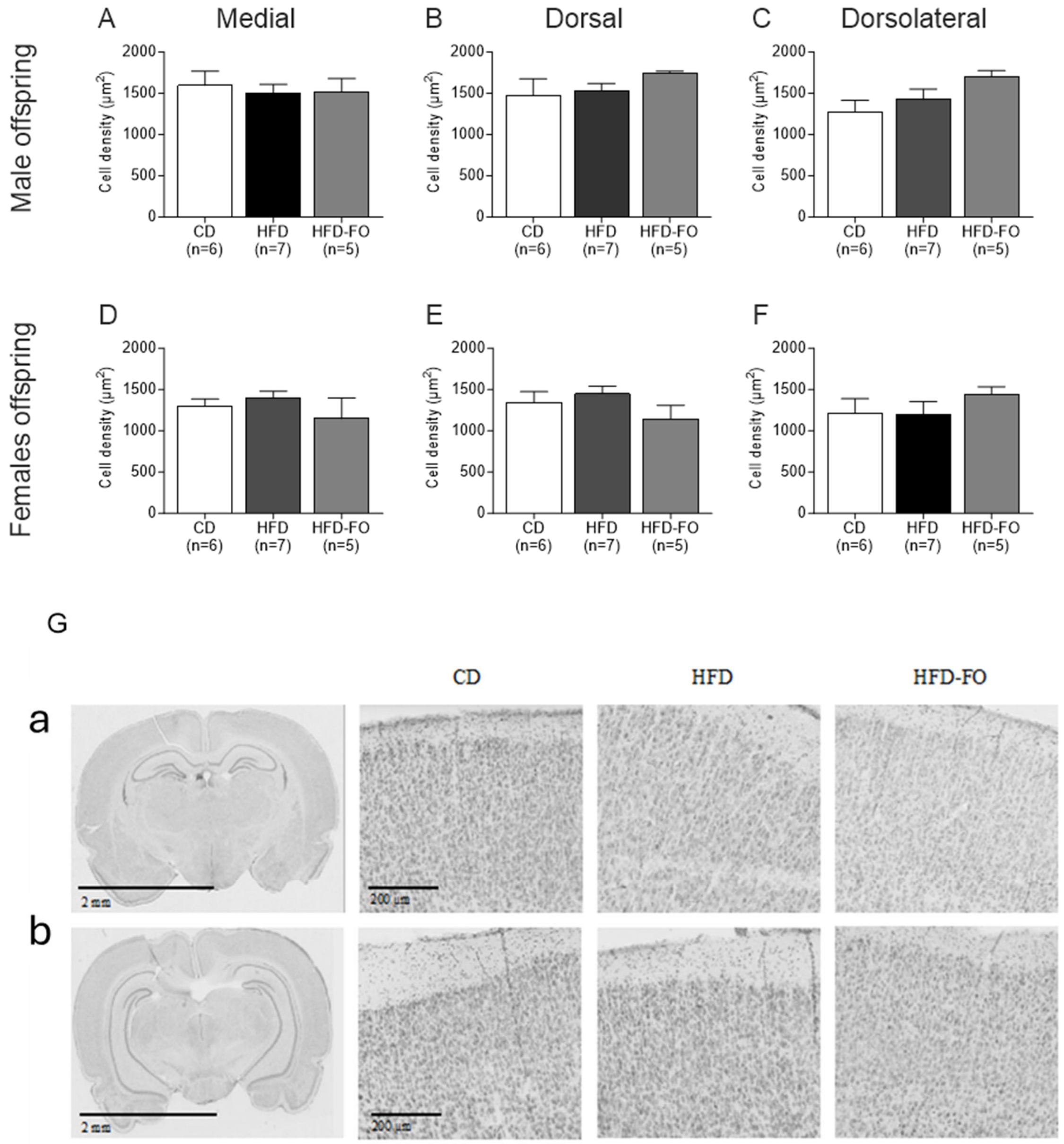

Figure S5. Neuron density in medial, dorsal, and dorsolateral areas of the brain cortex of male (A–C) and female (D–F) offspring's born to dams fed with control diet (CD), high-fat diet (HFD) and HFD enriched with fish oil (HFD-FO). CD (n = 6 mice per sex), HFD (n = 7 mice per sex) and HFD-FO (n = 5 mice per sex). Box shows representative images of each experimental group in males and females (G). Data are expressed as mean ± SEM. Differences were calculated by one–way ANOVA followed by Tukey's post-test or Kruskal-Wallis analysis followed by Dunn's tests.

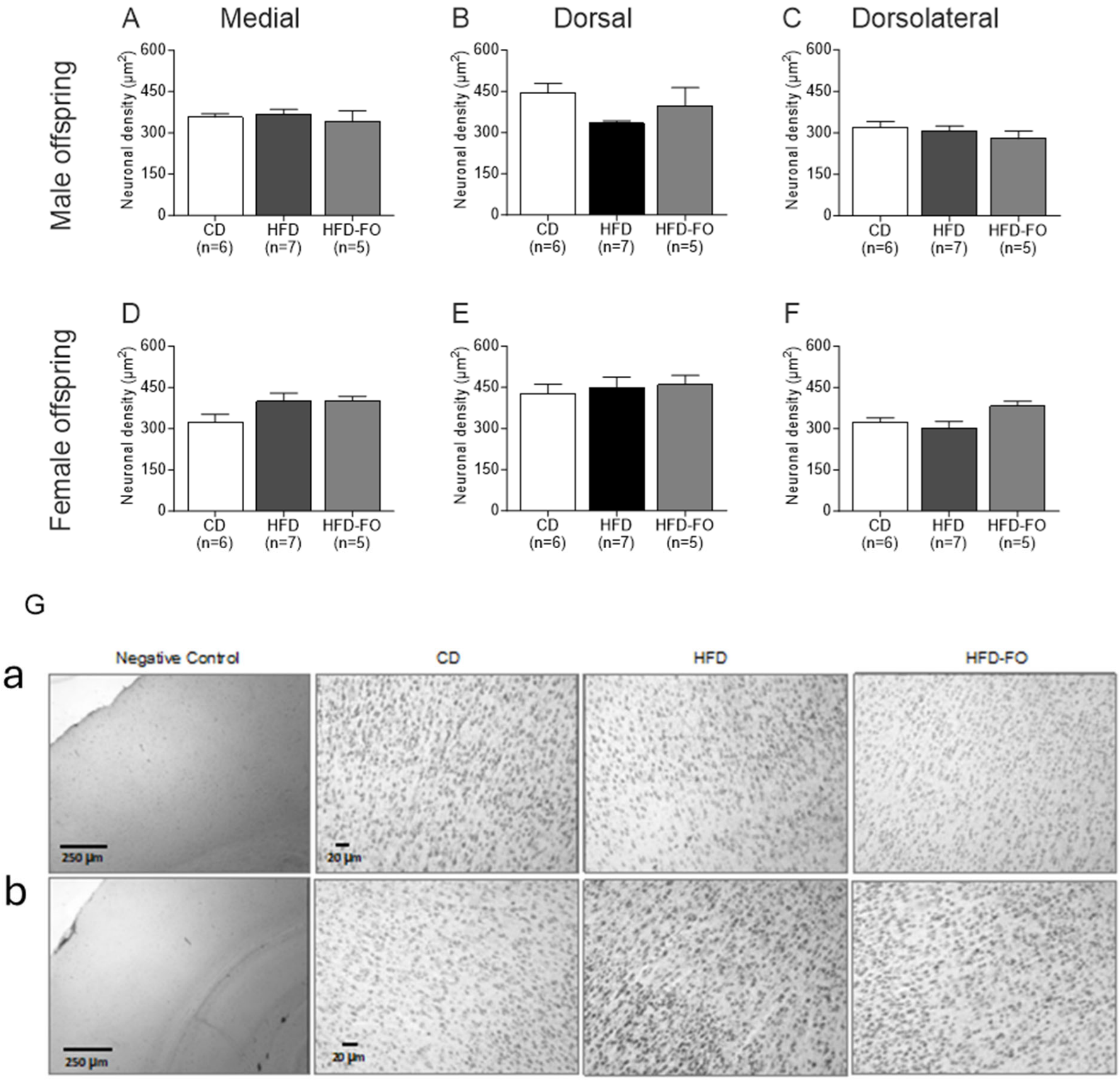

Supplement: Supplementary file 1 [file nutrients-17-01741-s001.zip › Supplemental Figures.pdf]
