# Supplementary material for: Fish Oil Supplementation Attenuates Offspring’s Neurodevelopmental Changes Induced by a Maternal High-Fat Diet in a Rat Model
Source: Nutrients. 2025 May 21;17(10):1741. doi: 10.3390/nu17101741 (PMC12113741; doi:10.3390/nu17101741)
Supplement: Supplementary file 1 [file nutrients-17-01741-s001.zip › Supplemental Tables.pdf]

SUPPLEMENTARY TABLES.

Table S1. Composition of fish oil (Menhaden fish oil) incorporated in the high-fat diet.

Component Name	Normalized by Weight %	%Triglyceride in Product
**12:0 Lauric**	0.140%	0.130
13:0 Tridecanoic	--	--
**14:0 Myristic**	9.435%	8.764
14:1 t-Tetradecanoic	0.080%	0.074
14:1 Myristoleic	0.058%	0.054
15:0 Pentadecanoic	0.578%	0.537
15:1 Pentadecenoic	--	--
**16:0 Palmitic**	16.800%	15.605
16:1 t-Hexadecenoic	0.422%	0.392
16:1 Palmitoleic	13.067%	12.137
17:0 Margaric	0.394%	0.366
17:1 Margaroleic	0.194%	0.180
18:0 Stearic	2.808%	2.608
18:1 trans-Elaidic	0.087%	0.081
18:1 Oleic	11.055%	10.268
18:2 t-Octadecadienoic	0.320%	0.297
18:2 Linoleic	3.186%	2.959
20:0 Arachidic	0.149%	0.138
18:3 g-Linolenic	0.343%	0.319
18:3 t-Linolenic	0.186%	0.173
20:1 Gadoleic	1.205%	1.119
18:3 Linolenic	1.316%	1.222
21:0 Heneicosanoic	--	--
**18:2 conjugated-Linoleic**	--	--
**18:4 Octadecatetraenoic**	2.977%	2.765
20:2 Eicosadienoic	--	--
**22:0 Behenic**	0.080%	0.074
20:3 g-Eicosatrienoic	0.163%	0.151
22:1 Erucic	0.116%	0.108
20:3 Eicosatrienoic	0.126%	0.117
20:4 Arachiodonic	0.990%	0.920
23:0 Tricosanoic	--	--
**22:2 Docasadienoic**	1.391%	1.292
24:0 Lignoceric	--	--
**20:5 Eicosapentaenoic**	18.538%	17.219
24:1 Nervonic	0.305%	0.283
22:3 Docosatrienoic	--	--
**22:4 Docosatetraenoic**	0.164%	0.152
22:5 Docosapentaenoic	2.857%	2.654
22:6 Docosahexaenoic	10.472%	9.727
Totals	100.00%	92.89

Table S2. Gene primers analyzed in the cerebral cortex of rat pups.

Gen	Primers
Synaptosome-associated protein 25 (Snap25).	Forward: GTTGGATGAGCAAGGCGAAC
	Reverse: CCTGATTATTGCCCCAGGCT
Brain derived neurotrophic factor (Bdnf).	Forward: GGCTGACACTTTTGAGCACG
	Reverse: ATGTTTGCGGCATCCAGGTA
Subunit p50 of NF-κB (Nfkb1)	Forward: TGCTTACGGTGGGATTGCAT
	Reverse: GGCAATTGCTTCGGTCTTGG
Major facilitator superfamily domain containing 2 (Mfsd2)	Forward: GAGACACTGGTCACGTGCTT
	Reverse: TGGATCGCTGTGCCTATCAC
Interleukin-1β (Il1b)	Forward: TAGCAGCTTTCGACAGTGAGG
	Reverse: CTCCACGGGCAAGACATAGG
Peptidilprolil isomerasa A (Ppia).	Forward: CAGGATTCATGTGCCAGGGTG
	Reverse: AGA TGC CAG GAC CTG TAT GC

Table S3. Biometric parameters in PND22 males and females offspring.

	Males				Females			
	CD (n = 6)	HFD (n = 7)	HFD-FO (n = 5)	ANOVA *P*-value	CD (n = 6)	HFD (n = 7)	HFD-FO (n = 5)	ANOVA *P*-value
Liver (mg)	5097.9 ± 513.7	4422.1 ± 100.4[a]	4413.1 ± 181.2[b]	0.002	4970.2 ± 319.5	4402.8 ± 148.0[a]	4576.1 ± 184.8[b]	0.001
Adipose tissue								
Inguinal (mg)	401.0 ± 58.4	693.0 ± 137.7[a]	583.5 ± 135.8	0.001	525.7 ± 85,5	767.4 ± 124.2[a]	621.9 ± 139.9	0.005
Mesenteric (mg)	408.9 ± 33.2	524.7 ± 74.8[a]	531.7 ± 75.6[b]	0.007	420.7 ± 41.3	547.8 ± 75.0[a]	508.5 ± 101.6	0.024
Retroperitoneal (mg)	300.6 ± 130.8	695.4 ± 182.5[a]	480.2 ± 171.2	0.002	208.6 ± 85.5	494.4 ± 127.3[a]	321.8 ± 126.8	0.001

Data presented as mean ± SEM. SFA: Saturated fatty acids; MUFA: Monounsaturated fatty acids; PUFA: Polyunsaturated fatty acids. CD: Control diet; HFD: high fat diet and HFD-FO: HFD enriched with fish oil. Differences were calculated by one-way ANOVA analysis followed by Tukey's post-test or Kruskal-Wallis analysis followed by Dunn's tests. [a] $p < 0.05$ between CD and HFD; [b] $p < 0.05$ between CD v/s HFD-FO.

Table S4. Intersections of neuronal arborization in PND22 male offspring.

Intersections	Experimental groups			*P* value
	CD	HFD	HFD-FO	
15 µm	3.0 + 1.0	4.0 + 1.0[a]	4.0 + 1.0	0.030
20 µm	3.0 + 1.0	5.0 + 1.0[a]	4.0 + 1.0[b]	0.008
25 µm	3.0 + 1.0	5.0 + 1.0[a]	5.0 + 2.0[b]	0.011
30 µm	3.0 + 1.0	4.0 + 1.0	5.0 + 1.0[b]	0.024
40 µm	3.0 + 1.0	4.0 + 1.0[a]	4.0 + 1.0	0.034

Data presented as mean + SEM. Neurons: CD (n=13), HFD (n=13), HFD-FO (n=13). One-way ANOVA analysis followed by Tukey's post-test or Kruskal-Wallis analysis followed by Dunn's tests. [a] p <0.05 between CD v/s HFD; [b] p <0.05 between CD v/s HFD-FO.

Table S5. Determination of fatty acids by gas chromatography in prefrontal cerebral cortex in male and female offspring at PND22.

Fatty acids (%mmol)	Males CD (n = 5)	Males HFD (n = 5)	Males HFD-FO (n = 5)	Males ANOVA *P-value*	Females CD (n = 5)	Females HFD (n = 5)	Females HFD-FO (n = 5)	Females ANOVA *P-value*
SFA								
C14:0	14.2 ± 1.7	14.3 ± 5.6	16.6 ± 4.8	0.649	15.3 ± 2.5	14.5 ± 2.0	13.8 ± 4.3	0.734
C15:0	0.4 ± 0.03	0.3 ± 0.05^{a}	0.4 ± 0.01	**0.022**	0.4 ± 0.1	0.3 ± 0.04^{a,c}	0.4 ± 0.03	**0.005**
C16:0	23.9 ± 0.4	20.2 ± 4.0	23.7 ± 1.2	0.200	21.6 ± 2.6	23.1 ± 1.2	24.5 ± 1.8	0.096
C18:0	10.3 ± 1.0	7.4 ± 3.7	11.5 ± 3.9	0.156	8.8 ± 0.6	9.8 ± 1.3	9.8 ± 2.4	0.536
ΣSFA	48.9 ± 1.3	42.4 ± 6.3	52.2 ± 8.2	0.069	46.0 ± 0.7	47.7 ± 2.0	48.5 ± 2.7	0.179
MUFA								
C14:1	0.9 ± 0.2	1.6 ± 0.7^{a}	0.9 ± 0.3	**0.030**	0.9 ± 0.3	1.0 ± 0.3	1.0 ± 0.3	0.562
C16:1 n-7	0.9 ± 0.1	0.7 ± 0.3	0.9 ± 0.1	0.164	0.8 ± 0.1	0.8 ± 0.1	0.8 ± 0.2	0.813
C18:1 n-9	19.7 ± 1.1	16.0 ± 3.4	17.5 ± 4.3	0.227	17.9 ± 3.0	19.2 ± 0.7	19.8 ± 1.1	0.294
C22:1 n-9	0.1 ± 0.1	0.2 ± 0.2	0.1 ± 0.03	0.089	0.3 ± 0.2	0.1 ± 0.1	0.2 ± 0.1	0.236
ΣMUFA	21.7 ± 1.1	18.6 ± 2.9	19.4 ± 4.2	0.274	19.8 ± 3.0	21.1 ± 0.7	21.8 ± 1.0	0.246
n-3 PUFA								
C18:3 n-3, ALA	0.1 ± 0.1	0.4 ± 0.2^{a}	0.2 ± 0.1^{c}	**0.001**	0.1 ± 0.04	0.1 ± 0.04	0.1 ± 0.03	0.412
C20:3 n-3	0.4 ± 0.1	0.2 ± 0.1	0.3 ± 0.1	0.142	0.2 ± 0.04	0.3 ± 0.1^{a}	0.3 ± 0.1	**0.036**
C20:5 n-3, EPA	0.4 ± 0.3	1.2 ± 0.5^{a}	0.2 ± 0.1^{c}	**0.017**	0.2 ± 0.1	0.3 ± 0.2	0.1 ± 0.02^{c}	**0.036**
C22:5 n-3, DPA n-3	0.7 ± 0.3	0.7 ± 0.3	0.6 ± 0.2	0.781	0.4 ± 0.1	0.6 ± 0.1	0.3 ± 0.2^{c}	**0.036**
C22:6 n-3, DHA	17.4 ± 1.4	26.5 ± 9.0	18.4 ± 4.7	0.062	24.4 ± 4.3	20.4 ± 1.4	20.4 ± 3.1	0.112
n-6 PUFA								
C18:2 n-6, LA	0.1 ± 0.1	1.3 ± 0.2^{a}	0.4 ± 0.4^{c}	**<0.001**	0.3 ± 0.1	0.2 ± 0.2	0.2 ± 0.1	0.543
C20:4 n-6, AA	9.6 ± 1.2	8.3 ± 1.4	7.6 ± 0.8	0.055	8.1 ± 1.4	8.8 ± 1.1	7.8 ± 1.7	0.562
C22:5 n-6, DPA n-6	0.7 ± 0.4	0.4 ± 0.4	0.8 ± 0.3	0.395	0.4 ± 0.2	0.5 ± 0.3	0.5 ± 0.1	0.738
ΣPUFA	29.4 ± 0.5	39.0 ± 9.1	28.4 ± 4.3^{c}	**0.026**	34.2 ± 3.1	31.2 ± 1.5	29.8 ± 2.9	0.052
Σn6 PUFA	10.4 ± 1.2	10.0 ± 1.4	8.8 ± 0.9	0.129	8.8 ± 1.6	9.6 ± 0.9	8.5 ± 1.6	0.487
Σn3 PUFA	19.0 ± 1.3	29.0 ± 9.1^{a}	19.6 ± 4.5	**0.034**	25.4 ± 4.3	21.7 ± 1.2	21.2 ± 3.1	0.111
Ratio (n6 : n3)	0.5 ± 0.1	0.4 ± 0.2	0.5 ± 0.2	0.198	0.4 ± 0.1	0.4 ± 0.05	0.4 ± 0.1	0.437

Data presented as mean ± SD. SFA: Saturated fatty acids; MUFA: Monounsaturated fatty acids; PUFA: Polyunsaturated fatty acids. CD: Control diet; HFD: high fat diet and HFD-FO: HFD enriched with fish oil. Differences were calculated by one-way ANOVA analysis followed by Tukey's post-test or Kruskal-Wallis analysis followed by Dunn’s tests . $^{a}p < 0.05$ between CD and HFD; $^{c}p < 0.05$ between HFD and HFD-FO.

Table S6. Determination of fatty acids by gas chromatography in motor cerebral cortex in male and female offspring at PND22.

Fatty acids (%mmol)	Males CD (n = 5)	Males HFD (n = 5)	Males HFD-FO (n = 5)	Males ANOVA *P-value*	Females CD (n = 5)	Females HFD (n = 5)	Females HFD-FO (n = 5)	Females ANOVA *P-value*
**SFA**								
C14:0	13.7 + 4.7	12.8 + 2.3	13.6 + 3.2	0.899	12.6 + 3.4	14.1 + 6.5	12.8 + 2.6	0.851
C15:0	0.4 + 0.1	0.3 + 0.04[a]	0.4 + 0.02	**0.008**	0.4 + 0.02	0.3 + 0.03[a,c]	0.4 + 0.03	**<0.001**
C16:0	24.1 + 2.9	22.3 + 4.0	25.9 + 1.9	0.223	25.0 + 2.3	25.8 + 2.2	24.9 + 1.4	0.728
C18:0	10.6 + 1.9	10.3 + 2.7	10.6 + 0.9	0.966	12.8 + 2.3	10.7 + 2.7	11.5 + 0.9	0.335
ΣSFA	48.7 + 1.5	45.7 + 4.2	50.6 + 4.5	0.151	50.8 + 1.2	50.9 + 2.1	49.5 + 1.0	0.323
**MUFA**								
C14:1	0.7 + 0.3	2.5 + 2.7	1.0 + 0.3	0.210	0.9 + 0.3	1.6 + 0.4	0.9 + 0.4	0.649
C16:1 n-7	0.9 + 0.2	0.8 + 0.2	1.0 + 0.2	0.156	1.0 + 0.1	0.7 + 0.1[a]	0.9 + 0.2	**0.026**
C18:1 n-9	20.1 + 2.6	19.3 + 1.3	21.7 + 1.5	0.175	19.4 + 1.8	20.7 + 1.6	20.4 + 1.3	0.401
C22:1 n-9	0.1 + 0.03	0.2 + 0.1	0.2 + 0.07[b]	**0.047**	0.1 + 0.1	0.1 + 0.03	0.1 + 0.1	0.561
ΣMUFA	21.8 + 2.8	22.8 + 1.7	23.8 + 1.5	0.357	21.4 + 1.7	22.6 + 1.4	22.3 + 1.1	0.394
**n-3 PUFA**								
C18:3 n-3, ALA	0.1 + 0.1	0.1 + 0.1	0.2 + 0.1	0.312	0.3 + 0.1	0.2 + 0.1[a]	0.2 + 0.1[b]	**0.003**
C20:3 n-3	0.4 + 0.2	0.3 + 0.1	0.4 + 0.03	0.188	0.3 + 0.02	0.4 + 0.1	0.4 + 0.1	0.650
C22:5 n-3, DPA n-3	0.1 + 0.1	0.5 + 0.5	0.7 + 0.2[b]	**0.024**	0.3 + 0.2	0.4 + 0.3	0.5 + 0.3	0.446
C20:5 n-3, EPA	0.4 + 0.2	1.6 + 0.5[a]	0.7 + 0.2[c]	**<0.001**	0.2 + 0.05	0.4 + 0.1	0.7 + 0.3[b,c]	**0.002**
C22:6 n-3, DHA	18.6 + 5.4	19.6 + 4.2	14.3 + 4.0	0.197	15.7 + 4.2	14.9 + 1.2	17.1 + 2.0	0.462
**n-6 PUFA**								
C18:2 n-6, LA	0.1 + 0.03	0.2 + 0.1	0.2 + 0.1	0.201	0.3 + 0.1	0.3 + 0.1	0.3 + 0.1	0.354
C20:4 n-6, AA	9.4 + 1.9	8.3 + 1.3	8.6 + 1.5	0.521	10.5 + 1.5	9.2 + 1.9	8.6 + 0.7	0.152
C22:5 n-6, DPA n-6	0.2 + 0.1	0.9 + 0.2[a]	0.7 + 0.2[b]	**<0.001**	0.3 + 0.04	0.8 + 0.03[a]	0.5 + 0.1[b,c]	**<0.001**
ΣPUFA	29.5 + 3.7	31.5 + 3.5	25.7 + 4.7	0.104	27.9 + 2.7	26.6 + 1.4	28.2 + 1.3	0.403
Σn6 PUFA	9.7 + 1.9	9.4 + 1.2	9.5 + 1.3	0.928	11.0 + 1.6	10.3 + 2.0	9.3 + 0.8	0.242
Σn3 PUFA	19.7 + 5.4	22.2 + 4.5	16.3 + 4.0	0.173	16.9 + 4.2	16.3 + 1.4	18.9 + 1.8	0.319
Ratio (n6 : n3)	0.5 + 0.2	0.5 + 0.2	0.6 + 0.1	0.426	0.7 + 0.3	0.6 + 0.2	0.5 + 0.1	0.217

Data presented as mean ± SD. SFA: Saturated fatty acids; MUFA: Monounsaturated fatty acids; PUFA: Polyunsaturated fatty acids. CD: Control diet; HFD: high fat diet and HFD-FO: HFD enriched with fish oil. Differences were calculated by one-way ANOVA analysis followed by Tukey's post-test or Kruskal-Wallis analysis followed by Dunn's tests . [a] $p < 0.05$ between CD and HFD; [b] $p < 0.05$ between CD v/s HFD-FO and [c] $p < 0.05$ between HFD and HFD-FO.

Table S7. Determination of fatty acids by gas chromatography in auditory cerebral cortex in male and female offspring at PND22.

	Males				Females			
Fatty acids (%mmol)	CD (n = 5)	HFD (n = 5)	HFD-FO (n = 5)	ANOVA *P-value*	CD (n = 5)	HFD (n = 5)	HFD-FO (n = 5)	ANOVA *P-value*
SFA								
C14:0	18.9 ± 4.5	17.9 ± 2.2	20.4 ± 3.8	0.568	13.3 ± 3.2	21.3 ± 2.6[a]	20.0 ± 6.7	**0.035**
C15:0	0.4 ± 0.1	0.3 ± 0.1[a]	0.3 ± 0.03[b]	**0.013**	0.4 ± 0.1	0.3 ± 0.03[a]	0.3 ± 0.1	**0.002**
C16:0	19.8 ± 4.5	20.2 ± 3.0	20.6 ± 3.0	0.925	23.5 ± 2.0	18.2 ± 2.02[a]	18.4 ± 3.6[b]	**0.013**
C18:0	7.8 ± 0.9	9.1 ± 1.7	7.0 ± 1.7	0.112	10.9 ± 1.7	7.0 ± 3.6	7.0 ± 2.5	0.062
ΣSFA	46.8 ± 2.9	47.5 ± 2.8	48.3 ± 5.1	0.826	48.1 ± 1.4	46.8 ± 3.1	45.7 ± 3.3	0.399
MUFA								
C14:1	0.9 ± 0.1	1.5 ± 0.2[a]	1.3 ± 0.3	**0.007**	0.8 ± 0.2	1.4 ± 0.1[a]	1.6 ± 0.1[b]	**< 0.001**
C16:1 n-7	0.7 ± 0.1	0.9 ± 0.3	0.5 ± 0.3	0.062	0.9 ± 0.1	0.4 ± 0.3[a]	0.6 ± 0.2[b]	**0.005**
C18:1 n-9	15.7 ± 4.4	16.5 ± 2.6	14.9 ± 3.9	0.802	19.3 ± 2.7	14.1 ± 1.0[a]	15.1 ± 3.5	**0.018**
C22:1 n-9	0.2 ± 0.1	0.2 ± 0.1	0.1 ± 0.05	0.355	0.1 ± 0.1	0.2 ± 0.1	0.2 ± 0.2	0.424
ΣMUFA	17.5 ± 4.5	18.9 ± 2.4	16.7 ± 4.3	0.657	21.2 ± 2.6	16.0 ± 1.2[a]	17.4 ± 3.7	**0.029**
n-3 PUFA								
C18:3 n-3, ALA	0.2 ± 0.03	0.09 ± 0.01[a]	0.1 ± 0.04	**0.023**	0.2 ± 0.1	0.1 ± 0.04	0.1 ± 0.03	0.295
C20:3 n-3	0.4 ± 0.1	0.3 ± 0.1	0.3 ± 0.2	0.479	0.5 ± 0.2	0.5 ± 0.2	0.3 ± 0.1	0.088
C22:5 n-3, DPA n-3	0.6 ± 0.2	0.1 ± 0.1[a]	1.2 ± 0.2[b,c]	**< 0.001**	0.1 ± 0.02	0.5 ± 0.1[a]	0.1 ± 0.03[c]	**< 0.001**
C20:5 n-3, EPA	0.6 ± 0.1	0.6 ± 0.4	0.6 ± 0.2	0.956	0.3 ± 0.2	0.9 ± 0.5	1.5 ± 0.6[b]	**0.004**
C22:6 n-3, DHA	26.2 ± 7.4	24.4 ± 3.7	25.2 ± 2.1	0.859	18.9 ± 4.6	29.2 ± 6.7[a]	27.9 ± 4.1[b]	**0.018**
n-6 PUFA								
C18:2 n-6, LA	0.2 ± 0.03	0.3 ± 0.01[a]	0.3 ± 0.05[b]	**< 0.001**	0.3 ± 0.1	0.2 ± 0.04[a]	0.2 ± 0.1[b]	**0.001**
C20:4 n-6, AA	7.3 ± 1.0	7.2 ± 1.2	6.5 ± 0.6	0.387	10.2 ± 1.7	5.7 ± 2.3[a]	5.8 ± 2.3[b]	**0.008**
C22:5 n-6, DPA n-6	0.5 ± 0.3	0.5 ± 0.4	0.9 ± 0.4	0.160	0.3 ± 0.2	0.2 ± 0.01	1.1 ± 0.3[b,c]	**< 0.001**
ΣPUFA	35.8 ± 6.4	33.6 ± 4.1	35.0 ± 2.4	0.748	30.7 ± 3.3	37.2 ± 4.1[a]	36.9 ± 3.8	**0.030**
Σn6 PUFA	7.9 ± 1.1	8.0 ± 1.2	7.7 ± 0.7	0.857	10.8 ± 1.7	6.0 ± 2.3[a]	7.1 ± 2.1[b]	**0.007**
Σn3 PUFA	27.9 ± 7.1	25.6 ± 3.7	27.4 ± 2.1	0.727	20.0 ± 4.9	31.2 ± 6.3[a]	29.9 ± 4.6[b]	**0.011**
Ratio (n6 : n3)	0.3 ± 0.1	0.3 ± 0.1	0.3 ± 0.03	0.737	0.6 ± 0.2	0.2 ± 0.1[a]	0.3 ± 0.1[b]	**0.003**

Data presented as mean ± SD. SFA: Saturated fatty acids; MUFA: Monounsaturated fatty acids; PUFA: Polyunsaturated fatty acids. CD: Control diet; HFD: high fat diet and HFD-FO: HFD enriched with fish oil. Differences were calculated by one-way ANOVA analysis followed by Tukey's post-test or Kruskal-Wallis analysis followed by Dunn's tests . [a] $p < 0.05$ between CD and HFD; [b] $p < 0.05$ between CD v/s HFD-FO and [c] $p < 0.05$ between HFD and HFD-FO.

Table S8. Determination of fatty acids by gas chromatography in liver in male and female offspring at PND22.

Fatty acids (%mmol)	Males				Females			
	CD (n = 5)	HFD (n = 5)	HFD-FO (n = 5)	ANOVA *P-value*	CD (n = 5)	HFD (n = 5)	HFD-FO (n = 5)	ANOVA *P-value*
SFA								
C14:0	1.1 ± 0.3	0.6 ± 0.2^{a}	1.0 ± 0.2^{c}	**0.001**	1.3 ± 0.4	0.7 ± 0.2^{a}	0.8 ± 0.2^{b}	**0.006**
C15:0	2.6 ± 1.1	0.6 ± 0.1^{a}	1.0 ± 0.6	**0.010**	3.2 ± 1.2	0.5 ± 0.2^{a}	0.7 ± 0.1^{b}	**<0.001**
C16:0	24.9 ± 1.3	21.9 ± 0.5^{a}	22.3 ± 0.6^{b}	**<0.001**	25.8 ± 1.5	21.7 ± 1.3^{a}	22.8 ± 0.7^{b}	**<0.001**
C18:0	13.9 ± 2.9	20.0 ± 2.2^{a}	14.1 ± 1.9^{c}	**<0.001**	12.8 ± 1.9	17.9 ± 3.7^{a}	15.3 ± 0.6	**0.010**
C20:0	0.3 ± 0.1	0.3 ± 0.1	0.4 ± 0.1^{c}	**0.027**	0.2 ± 0.1	0.3 ± 0.03	0.5 ± 0.1^{b,c}	**<0.001**
C22:0	0.5 ± 0.2	0.5 ± 0.1	0.5 ± 0.1	0.935	0.5 ± 0.1	0.4 ± 0.2	0.5 ± 0.1	0.175
**ΣSFA**	43.3 ± 4.0	43.8 ± 2.5	39.2 ± 1.9^{c}	**0.042**	43.7 ± 3.6	41.4 ± 3.6	40.6 ± 1.1	0.247
MUFA								
C14:1	0.3 ± 0.1	0.1 ± 0.04^{a}	0.2 ± 0.04	**0.008**	0.3 ± 0,1	0.2 ± 0.2	0.2 ± 0.1	0.603
C16:1 n-7	2.9 ± 1.0	0.9 ± 0.3^{a}	1.9 ± 0.5^{c}	**<0.001**	3.7 ± 0,7	0.9 ± 0.1^{a}	1.8 ± 0.2	**<0.001**
C18:1 n-9	22.7 ± 5.3	24.7 ± 3.4	14.2 ± 4.4^{b,c}	**0.002**	25.5 ± 4,6	25.9 ± 3.8	13.1 ± 1.0^{b,c}	**<0.001**
**ΣMUFA**	26.0 ± 6.1	25.8 ± 3.6	16.3 ± 4.9^{b,c}	**0.007**	29.5 ± 5,0	27.1 ± 3.9	15.1 ± 1.0^{b,c}	**0.002**
n-3 PUFA								
C18:3 n-3, ALA	0.2 ± 0.1	0.1 ± 0.04	0.1 ± 0.1	0.380	0,2 ± 0,1	0.1 ± 0.04	0.1 ± 0.1	0.058
C20:3 n-3	0.2 ± 0.1	0.4 ± 0.1^{a}	0.2 ± 0.02^{c}	**<0.001**	0,2 ± 0,1	0.4 ± 0.04^{a}	0.2 ± 0.01^{c}	**<0.001**
C22:5 n-3, DPA n-3	0.3 ± 0.2	0.1 ± 0.1	0.1 ± 0.03	0.515	0,1 ± 0,1	0.3 ± 0.3	0.2 ± 0.1	0.362
C20:5 n-3, EPA	0.6 ± 0.6	0.3 ± 0.3	0.4 ± 0.2	0.432	0,6 ± 0,4	0.4 ± 0.3	0.8 ± 0.3	0.110
C22:6 n-3, DHA	7.8 ± 1.4	4.9 ± 0.8^{a}	8.6 ± 0.8^{c}	**<0.001**	6,0 ± 1,6	4.2 ± 1.4	8.1 ± 1.0^{b,c}	**<0.001**
n-6 PUFA								
C18:2 n-6, LA	9.7 ± 1,2	12.4 ± 2.5	14.5 ± 3.0^{b}	**0.011**	9,9 ± 2,4	13.9 ± 3.0^{a}	15.3 ± 1.1^{b}	**0.005**
C20:4 n-6, AA	9.2 ± 2,9	10.1 ± 2.3	14.5 ± 1.0^{b,c}	**0.003**	8,3 ± 2,7	10.0 ± 1.1	13.4 ± 1.0^{b,c}	**<0.001**
C22:5 n-6, DPA n-6	2.8 ± 1,5	2.0 ± 0.9	6.0 ± 1.3^{b,c}	**<0.001**	1,5 ± 0,3	2.3 ± 1.8	6.2 ± 1.6^{b,c}	**<0.001**
**ΣPUFA**	30.7 ± 5.8	30.4 ± 5.9	44.5 ± 3.3^{b,c}	**<0.001**	26,8 ± 6,6	31.5 ± 3.0	44.3 ± 1.1^{b,c}	**<0.001**
**Σn6 PUFA**	21.7 ± 5.1	24.6 ± 5.3	35,1 ± 3.4^{b,c}	**<0.001**	19,7 ± 5,2	26.2 ± 3.3^{a}	34.9 ± 1.0^{b,c}	**<0.001**
**Σn3 PUFA**	9.0 ± 1.2	5.8 ± 0.9^{a}	9.5 ± 0.6^{c}	**<0.001**	7,1 ± 1,8	5.4 ± 0.9	9.5 ± 1.1^{b,c}	**<0.001**
Ratio (n6 : n3)	2.4 ± 0.5	4.2 ± 0.7^{a}	3.7 ± 0.5^{b}	**<0.001**	2,9 ± 0,5	5.0 ± 1.3^{a}	3.7 ± 0.5	**0.002**

Data presented as mean ± SD. SFA: Saturated fatty acids; MUFA: Monounsaturated fatty acids; PUFA: Polyunsaturated fatty acids. CD: Control diet; HFD: high fat diet and HFD-FO: HFD enriched with fish oil. Differences were calculated by one-way ANOVA analysis followed by Tukey's post-test or Kruskal-Wallis analysis followed by Dunn’s tests . $^{a}p < 0.05$ between CD and HFD; $^{b}p < 0.05$ between CD v/s HFD-FO and $^{c}p < 0.05$ between HFD and HFD-FO.
